# A new iron-phosphate compound (Fe_7_P_11_O_38_) obtained by pyrophosphate stoichiometric glass devitrification

**DOI:** 10.1038/s41598-021-02471-0

**Published:** 2021-11-25

**Authors:** Pawel Goj, Aleksandra Wajda, Artur Błachowski, Pawel Stoch

**Affiliations:** 1 AGH-University of Science and Technology, Faculty of Materials Science and Ceramics, Al. Mickiewicza 30, 30-059 Kraków, Poland; 2 Jagiellonian University, Faculty of Chemistry, Gronostajowa 2, 30-387 Kraków, Poland; 3 Mössbauer Spectroscopy Laboratory, Institute of Physics, Pedagogical University, ul. Podchorążych 2, 30-084 Kraków, Poland

**Keywords:** Solid-state chemistry, Magnetic materials, Electronic materials, Electronic structure

## Abstract

Iron phosphates are a wide group of compounds that possess versatile applications. Their properties are strongly dependent on the role and position of iron in their structure. Iron, because of its chemical character, is able to easily change its redox state and accommodate different chemical surroundings. Thus, iron-phosphate crystallography is relatively complex. In addition, the compounds possess intriguing magnetic and electric properties. In this paper, we present crystal structure properties of a newly developed iron-phosphate compound that was obtained by devitrification from iron-phosphate glass of pyrophosphate stoichiometry. Based on X-ray diffraction (XRD) studies, the new compound (Fe_7_P_11_O_38_) was shown to adopt the hexagonal space group P6_3_ (No. 173) in which iron is present as Fe^3+^ in two inequivalent octahedral and one tetrahedral positions. The results were confirmed by Raman and Mössbauer spectroscopies, and appropriate band positions, as well as hyperfine interaction parameters, are assigned and discussed. The magnetic and electric properties of the compound were predicted by ab initio simulations. It was observed that iron magnetic moments are coupled antiferromagnetically and that the total magnetic moment of the unit cell has an integer value of 2 µ_B_. Electronic band structure calculations showed that the material has half-metallic properties.

## Introduction

Iron-phosphate compounds, as well as glasses (IPG) are important materials, which can find applications in different fields such as biomedical and electrical devices, waste immobilization, optical instruments, etc^1–4^. Their properties depend on the structure of the phosphate network and the role and atomic position of iron. Moreover, iron may very strongly improve the chemical durability. Thus, it is possible to obtain a material with superior water resistance that may be used in waste immobilization processes^5,6^. On the other hand, controlled glass crystallization may lead to the achievement of materials with even better properties. Appropriate crystalline phases can incorporate specific waste components that are additionally protected by the residual glassy phase. As a consequence, multibarrier material with enhanced immobilization properties of waste components can be obtained. Controlled glass crystallization is not an easy task and depends on many factors such as glass composition, heat treatment procedure, synthesis method, reagents etc^7^.

In the case of IPG, the problem of proper crystallization is even more complicated, as glass may contain iron in both Fe^2+^ and Fe^3+^ valences. The amount of Fe^2+^ depends mainly on the preparation conditions^8^. This leads to a very complex crystallography of the FeO-Fe_2_O_3_-P_2_O_5_ system with more than 20 different crystalline iron-phosphate phases present at ambient pressure^9^. In which iron can be present in both valences, different coordination to oxygen (4–6), and some of them like FePO_4_ may exist in several polymorphic forms^9,10^. The possibility of changing the valence state by iron ions gives them an easier way to accommodate different crystallochemical surroundings and makes them more flexible in adopting the optimal charge to ensure charge neutrality or chemical bond ionicity^11–13^.

One of the most promising from a waste treatment perspective is IPG glass of compositions 40Fe_2_O_3_60P_2_O_5_ (pyrophosphate stoichiometry). The high amount of Fe_2_O_3_ ensures superior water durability. The glass composition is a compromise between the durability and thermal stability of the glass. The higher concentration of Fe_2_O_3_ can lead to partial and uncontrolled crystallization of the vitreous phase. Glass devitrification was evidenced to lead to the formation of two main crystalline compounds Fe_3_(P_2_O_7_)_3_ and Fe_4_(P_2_O_7_)_3_^14^. However, other compounds such as FePO_4_^15^, Fe_2_P_2_O_7_^16^, Fe(PO_3_)_3_^17^ cannot be excluded. It was also observed that glass crystallization is a surface nucleated process in which mixed-valence iron Fe_3_(P_2_O_7_)_3_ is oxidized and transformed into Fe_4_(P_2_O_7_)_3_ and FePO_4_. The process follows from the surface to the bulk of the material, and FePO_4_ can be detected only on surface^18^.

The structural features, differences in valency, disorder, etc. of iron-phosphates may lead to different spin sublattices and interesting magnetic properties. In the group of materials, antiferromagnetic coupling of iron magnetic moments is frequently reported. To such compounds belong e.g. Fe_2_P_2_O_7_, Fe_3_PO_7_, Fe_3_(P_2_O_7_)_2_, Fe_4_(P_2_O_7_)_3_, (NH_4_)Fe_2_(PO_4_)_2_, where depending on the compound the coupling may be realized via direct interaction (Fe–Fe), superexchange (Fe–O–Fe), super-superexchange (Fe–O–P–O–Fe) mechanism^19–22^. However, the coupling is relatively weak and the magnetic ordering temperatures are also low. Even more intriguing are IPG glasses that, despite the lack of structural order, can exhibit magnetic ordering and spin glass-like antimagnetic behavior^9,10,23–26^.

The electrical properties of iron phosphates are also very curious. The materials have semiconducting features with an electronic conduction mechanism that occurs through electron hopping between Fe^2+^ and Fe^3+^ ions. The typical ionic component of electrical conductivity is much lower than the electronic one^27,28^.

The work describes structural and hyperfine features, as well as, electric and magnetic properties predicted based on ab initio simulations of a new iron-phosphate compound obtained during devitrification of 40Fe_2_O_3_60P_2_O_5_ glass.

## Results and discussion

### XRD analysis

**Figure 1:**
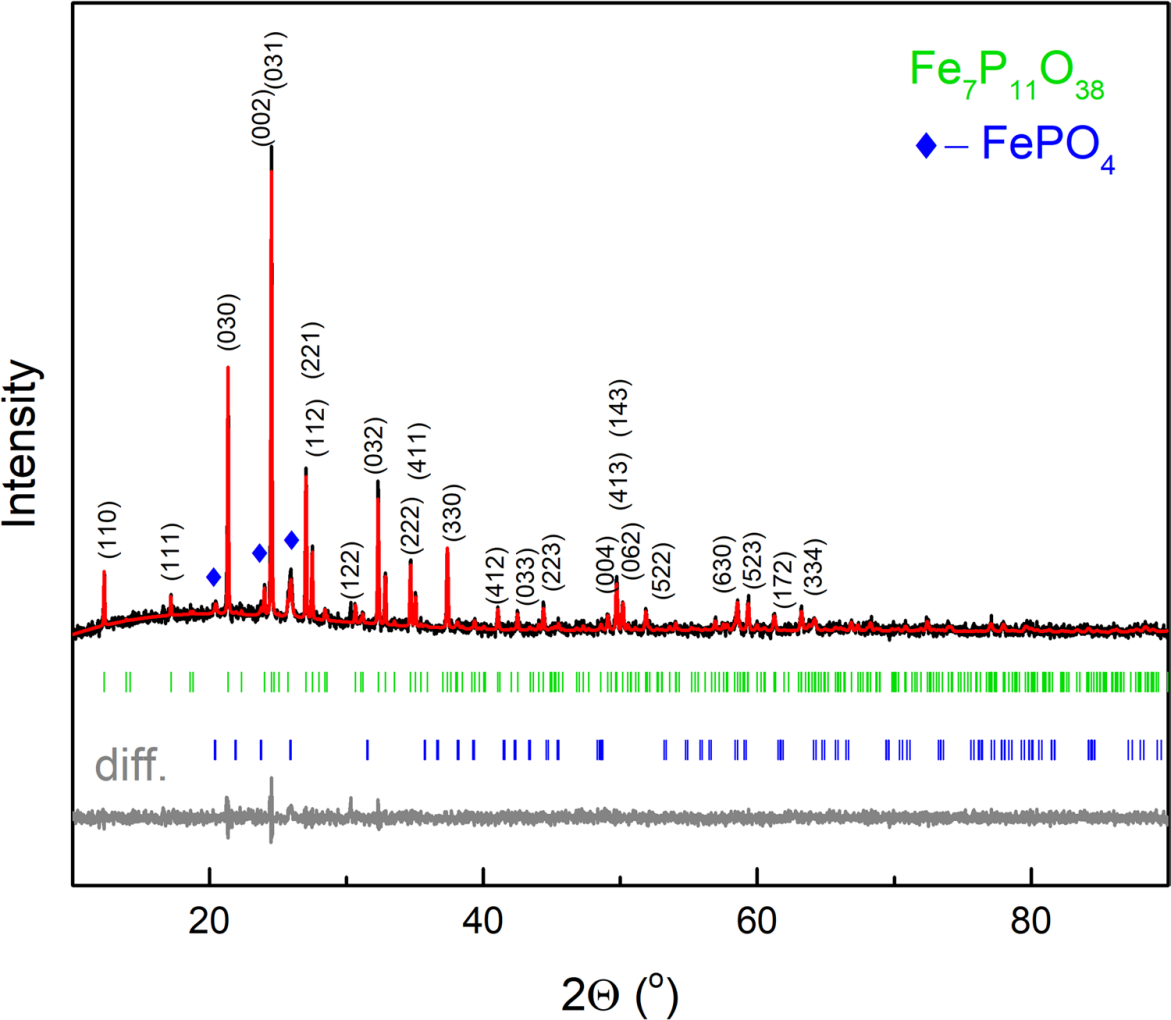
Measured X-ray diffractogram (black) of the crystalized sample (new phase Fe_7_P_11_O_38_ and traces of FePO_4_) with fitted (red) and differential (gray) patterns. Green and blue ticks in the figure represent theoretical positions of Bragg peaks for Fe_7_P_11_O_38_ and FePO4, respectively. The most intense Bragg peaks of FePO_4_ are marked with blue marks.

**Figure 2:**
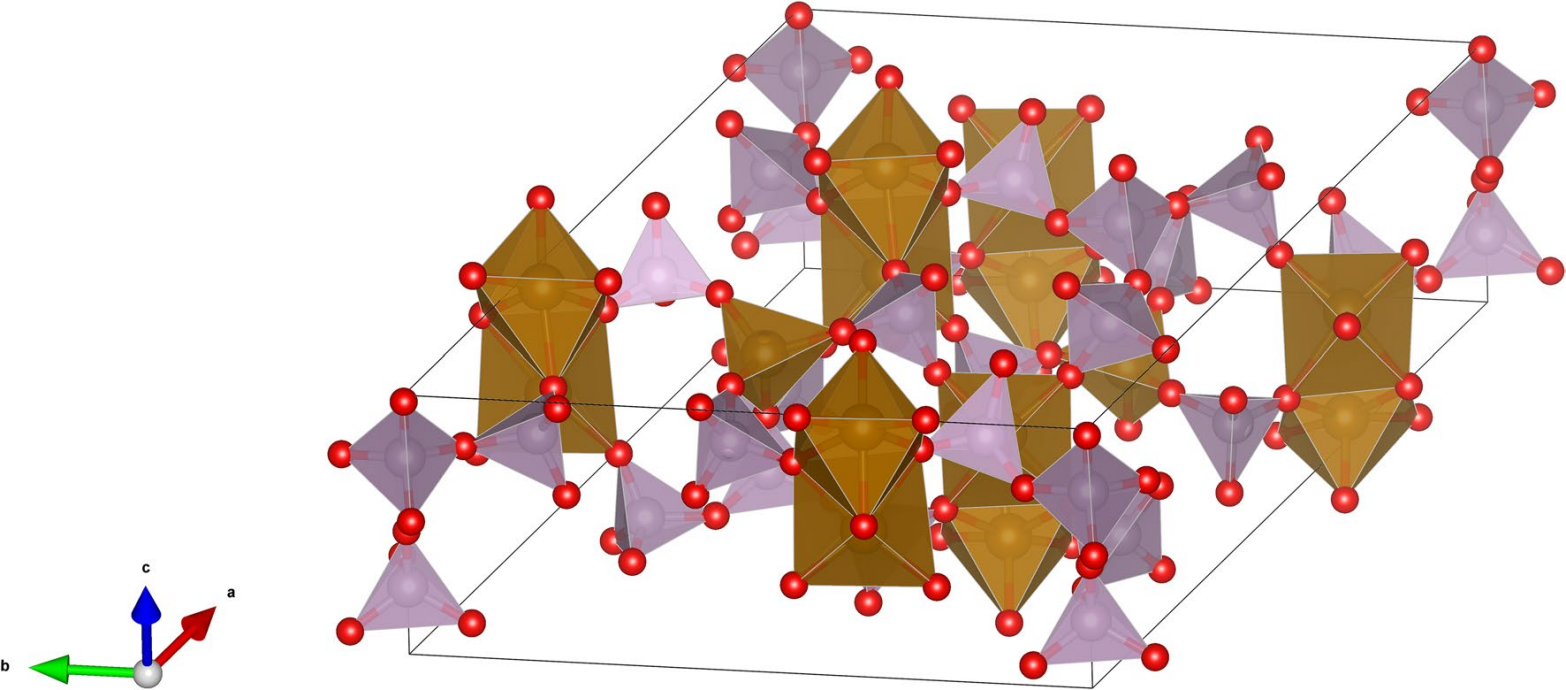
The new Fe_7_P_11_O_38_ compound (oxygens are marked in red, phosphorus tetrahedra are purple, and iron polyhedrons are brown).

The XRD pattern of the devitrified material (Fig. 1) is characterized by intense reflections of the crystalline compounds and a relatively low background with a characteristic broad halo around 2Θ≈20° that may originate from the residual glassy phase. First, the obtained pattern was quantitatively analyzed by comparing it with XRD patterns from databases (COD, AMMIN, ICCD, PDF-4). There are several reflections that match rodolicoite (berlinite-type FePO_4_) marked in Fig. 1 as rhombus. However, the positions and intensities of the main intense peaks did not match any iron-phosphate phase in the databases. Furthermore, based on structural refinement using the EXPO2014 program^29^ it was detected that the unknown compound has a hexagonal symmetry and may belong to the P6_3_/m or P6_3_ space groups. Comparison of the positions and intensities of the peaks of other hexagonal symmetry phosphate compounds showed that the pattern is similar to vanadosilicophosphate (V_3_P_5_SiO_19_)^30^ of the P6_3_ space group. Since in the devitrified material there is no Si nor V, the atoms were replaced by P and Fe in the following way. All V^3+^ ions were replaced by Fe^3+^ in their structural positions. Since in the structure there are two inequivalent tetrahedral Si sites, the [SiO_4_] tetrahedra are joined by a common bridging oxygen. The other oxygen atoms create joints with [PO_4_] tetrahedra. Thus, one Si^4+^ ion was replaced by P^5+^ and the other by Fe^3+^. In this way, the [PO_4_]^+^ tetrahedron is joined to the [FeO_4_]^-^ tetrahedron to ensure the charge neutrality of the net. The similar connection is characteristic for e.g. rodolicoite. Furthermore, the crystal structure parameters and atomic positions were fitted with least squares together with the basic structural parameters of the secondary rodolicoite phase. The fitted (red) and differential patterns (gray) are presented in Fig. 1. The refinement led to χ^2^ = 1.25, R_w_ = 0.025. The unit cell parameters of the new compound (Fe_7_P_11_O_38_) of the hexagonal P6_3_ space group No. 173 are a = b = 14.4504(16) Å, c = 7.4261(2) Å. The weight fraction of the main compound is 89.5%. It should be noted that the phase stoichiometry (Fe/P = 0.64) is very close to the glass (Fe/P = 0.67). The detailed atom positions, interatomic distances, selected angles, and peaks list are given in the supplementary material (Tables S1–S4), as well as corresponding structural CIF file. The unit cell of the phase is shown in Fig. 2.

The main building elements of the structure are [PO_4_] tetrahedrons, [FeO_6_] octahedrons that have a common face and create [Fe_2_O_9_] dimers, and [FeO_4_] tetrahedrons. For the two phosphorous atoms in the Wycoff 6c positions, the [PO_4_] tetrahedrons share three corners with different [Fe_2_O_9_] dimers and one with [PO_4_], where the phosphorus is at the 2b site. Thus, the [PO_4_] tetrahedrons have one bridging oxygen and may be classified as Q^1^ structural units. There is also another phosphorus atom at the 6c position that shares three corners with [Fe_2_O_9_] and one with [FeO_4_], where iron is in the 2b site. Thus, the [PO_4_] tetrahedrons do not have bridging oxygen atoms (Q^0^). For P at 2b sites, the tetrahedrons share three corners with [PO_4_] and one with [FeO_4_]. Therefore, the units have three bridging oxygen atoms (Q^3^). There are also P atoms in 2a sites for which the oxygen tetrahedrons are Q^3^ structural units, the fourth oxygen atom creates the P=O double bond. Therefore, in the unit cell, there are 4Q^3^, 12Q^1^, and 6Q^0^ phosphate structural units. Iron atoms occupy two inequivalent octahedral sites (6c) and one tetrahedral (2b).

The [PO_4_] tetrahedrons are rather regular, however, the phosphorus atoms are slightly off-centered due to the distribution of the P-O distances. The longest P–O distance is observed for bridging oxygen atoms and it is in the range of 1.56–1.60 Å. The shorter P–O distance occurs when the oxygen atom is common to iron in tetrahedral sites in the range of 1.52–1.54 Å. The shortest P-O distance, in the range of 1.43–1.52 Å, is when oxygen atoms are common for two [FeO_6_] octahedrons. The observed values and their distributions are in agreement with the results for similar iron-phosphates^21,30^, as well as those theoretically predicted in iron-phosphate glasses^11,12,31^.

In the crystal structure, iron atoms occupy three different sites. One tetrahedral (2b) and two inequivalent octahedral (6c) positions. For iron at the 2b site, the Fe–O distance is in the range of 1.72–1.80 Å, while in the case of octahedral sites the distance is higher, in the range of 1.83–2.23 Å. The volume of [FeO_4_] is 2.650 Å^3^ with the bond length distortion index of 0.0061. The volumes of the [FeO_6_] polyhedrons are 10.590 Å^3^, 10.902 Å^3^ and correspond to the distortion indexes 0.0620, 0.0565.

The secondary phase is a FePO_4_ berlinite type that adopts the space group P3_1_21 (No 152). In the phase, iron atoms occupy 3a (0.4600, 0.000, 0.3333) sites, whereas phosphorous atoms are located in 3b (0.7000, 0.0000, 0.8333) sites. Both of the positions are tetrahedrally coordinated. The [PO_4_] and [FeO_4_] tetrahedrons are alternately joined and create 6 membered rings. The [PO_4_] tetrahedrons are positively charged, whereas the [FeO_4_] are negative. The alternative joining ensures the charge neutrality of the net. The parameters of the fitted crystal structure are a = b = 5.0273(4) Å, c = 11.2499(7) Å and they are in agreement with the reference data (COD-1518115).

### Mössbauer spectroscopy

**Figure 3:**
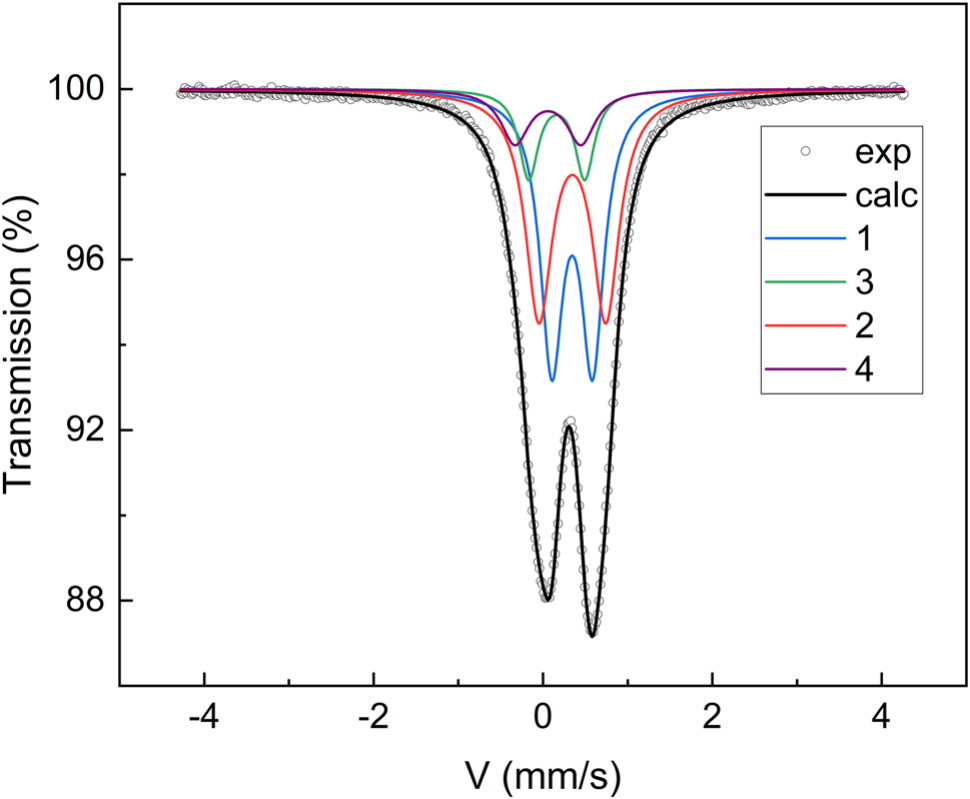
^57^Fe transmission Mössbauer effect spectrum of the devitrified glass containing Fe_7_P_11_O_38_ and traces of FePO_4_ at room temperature. The fitted subspectral components (1–4) are designated as in Table 1.

^57^Fe Mössbauer spectroscopy gives a unique possibility to look at the structure of the obtained material from an iron atoms perspective. The measured ^57^Fe Mössbauer transmission spectrum of the devitrified glass at room temperature is presented in Fig. 3.

The spectrum was least-squares fitted assuming that each inequivalent iron site individually contributes to the spectrum. In the material, there are three individual iron sites due to the new Fe_7_P_11_O_38_ compound (2 octahedral, 1 tetrahedral) and one tetrahedral iron site originating from FePO_4_. Thus, the spectrum was fitted assuming 4 components. At first glance, it can be evidenced that in the sample there is no Fe^2+^. Although iron is introduced as Fe^3+^ part of it is frequently reduced during iron-phosphate glass synthesis. The reduction level depends strongly on the synthesis conditions. According to our previous observations of the effect in iron-phosphate glass prepared similarly, the Fe^2+^ quantity is in the range of 15–30%^16,32,33^. The Mössbauer measurement for the glass sample (not shown here) revealed that in the glass about 15% of Fe^3+^ was reduced to Fe^2+^. Therefore, we may assume that the whole Fe^2+^ was oxidized during the crystallization process that was carried out in the air atmosphere.

The hyperfine interaction parameters fitted, namely the relative area of the subspectral component, the isomer shift related to α-Fe (IS), the quadrupole split (e^2^Q/2), and half-width at half maximum are given in Table 1.

**Table 1:** Hyperfine interactions parameters (A—relative area, IS—isomer shift, e^2^Q/2—quadrupole split, Γ—half-width at half maximum).

No	A (%)	IS (mm/s)	e^2^Q/2 (mm/s)	Γ (mm/s)	Assignment
1	39.6(1)	0.453(1)	0.482(1)	0.163(1)	^VI^Fe(III)
2	39.4(2)	0.456(1)	0.790(2)	0.194(2)	^VI^Fe(III)
3	9.8(3)	0.169(1)	0.780(7)	0.201(3)	^IV^Fe(III)
4	11.2(3)	0.271(2)	0.662(3)	0.138(2)	^IV^Fe(III)–FePO_4_

The values of the IS parameter confirmed that in the material all iron atoms are Fe^3+^ in a high-spin state and can be distinguished by two groups of the parameter. The lower IS values that are characteristic for Fe^3+^ in tetrahedral coordination (components 3, 4), and the higher due to Fe^3+^ in octahedral coordination (components 1, 2)^34–36^. According to the XRD results in the studied material, secondary α-FePO_4_ (rodolicoite) is detected. For the phase, IS values are relatively high like for a tetrahedrally coordinated Fe^3+^ and are about 0.3 mm/s, and e^2^Q/2 is c.a. 0.62 mm/s^37,38^. Therefore, the last component (No. 4) was assigned to α-FePO4, for which the IS value obtained is 0.271 mm/s and e^2^Q/2 = 0.662 mm/s. Assuming that the relative area of the component is proportional to the number of iron atoms involved in phase formation, we may conclude that 11.2% of iron atoms are in α-FePO_4_. On the basis of the XRD the weight quantity of the phase is about 10.5%. This means that approximately 13% of the iron atoms are in α-FePO_4_ following the Mössbauer result (11.2%).

The rest of the spectral components may be related to the new Fe_7_P_11_O_38_ compound. In the compound, two octahedral iron sites are equally populated. These are components number 1, 2 that have almost equal contribution and value of IS. Both sites have the same coordination, so the small difference may be due to a slight variation in the Fe–O distance or the octahedral volume. On the other hand, the IS, as well as the volume changes, are very small and maybe affect by the analysis uncertainty. Although the IS value is almost equal, the sites may be resolved based on the quadrupole split parameter. The parameter is sensitive to an electric charge distribution around an iron ion, and the higher the e^2^Q/2 value, the lower the symmetry of the site^34,39,40^. Thus, in the phase, there are two iron octahedral sites, one of a higher symmetry and the other of a lower symmetry or a higher distortion. This is in agreement with XRD, where the two iron positions have two different length distortion indexes.

In the unit cell, there are also tetrahedral iron positions that are 6 times less populated than the octahedral ones. The positions are described by spectral component No 3. The relative area of the component is smaller but comparable to the expected based on a simple population analysis of the sites. This may suggest that part of the iron positions may be occupied by phosphorus atoms. On the other hand, the minor α-FePO_4_ is richer in iron compared to glass stoichiometry. Its formation may result in a slight off-stoichiometry of the new phase that may promote the substitution of tetrahedral iron or part of the octahedral iron may be still in the vitreous phase. The second effect cannot be excluded taking into account observation of the residual amorphous halo in Fig. 1. The QS value for the site is also higher compared to the value for α-FePO_4_. This suggests a higher distortion of the position compared to that of the rodolicoite.

Thus, the Mössbauer spectroscopy results are consistent with the XRD studies.

### Raman spectroscopy

**Figure 4:**
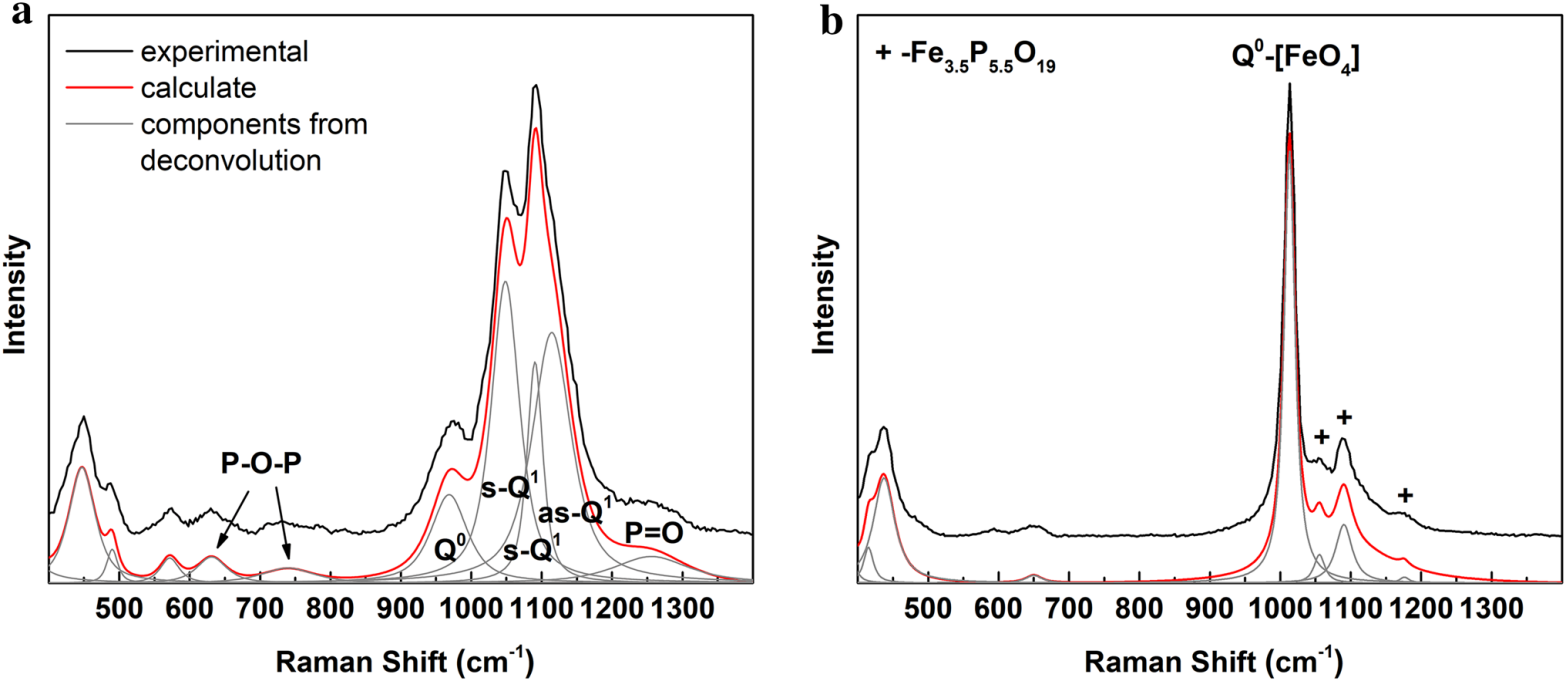
Raman spectra and their deconvolution of **(a)** Fe_7_P_11_O_38_ and **(b)** FePO_4_ dominating phases.

In this case, Raman spectroscopy gives the possibility to look into the material mostly from a phosphorus perspective. The spectra were measured at several points, and two types of different groups of spectra were distinguished. The spectra belonging to the same group were averaged. The averaged spectra from each group are shown in Fig. 4.

Most of the collected spectra (about 80%) belong to the first group (Fig. 4a). The average spectrum was fitted and the parameters of the fitted components are collected in Table 2. The obtained bands were assigned according to the literature^11,41–43^.

**Table 2:** Fitted Raman spectra of the components of Fig. 4^11,41–43^.

Crystalline Fe_7_P_11_O_38_				
No	Frequency (cm^−1^)	Area	Width (cm^−1^)	Assignment
1	1255	21.25	126.53	Stretching vibrations of P=O bonds
2	1114	105.59	66.59	Asymmetric stretching vibrations of P–O bonds in Q^1^
3	1090	37.74	26.96	Symmetric stretching vibrations of P–O bonds in Q^1^ in Q^1^-[FeO_6_]
4	1048	91.95	48.14	
5	968	34.74	62.10	Symmetric stretching vibrations of P–O bond in Q^0^
6	739	8.67	90.03	Symmetric stretching vibrations of P–O–P
7	631	8.41	50.57	Bending vibrations of P–O–P
8	571	5.15	32.43	Bending vibrations of P–O
9	490	3.97	18.47	
10	447	32.95	45.01	

In the case of phosphates, the most intense bands are located in the range of 900–1450 cm^−1^ and are assigned to symmetric stretching vibrations of P-O bonds in different Q^i^ structural units. The most intense bands are related to symmetric vibrations of Q^1^ units (bands No 3, 4), with these bands being associated bands of asymmetric vibrations (band No 2). However, the intensity of the band compared to the intensity of the symmetric vibrations is too high. This may suggest that in the band, the partial contribution of Q^2^ units cannot be excluded. The units are not observed in the crystalline compounds but may originate in the residual glassy phase. Except, the dominating bands associated with Q^1^ in the spectra there are observed less intense bands related to Q^3^ (band No 1) and Q^0^ (band No 5). When comparing the ratios of the specific bands, it can be seen that Q^3^/Q^0^ is approximately 0.61, and it is in very good agreement with the theoretical value of 0.6, which is the result of number of the units in the Fe_7_P_11_O_38_ unit cell. Similar ratios to Q^1^ are Q^3^/Q^1^ c.a. 0.16, and Q^0^/Q^1^ c.a. 0.27. The theoretical values are about twice as large for both cases. This may be related to the error of the analysis associated with the asymmetric vibrations, which overlap and cannot be included, or the higher number of Q^1^ units is embedded in the residual vitreous phase. However, taking into account the types of vibrations and approximate intensities, the spectrum can be assigned to Fe_7_P_11_O_38_. Additionally, the position of the Q^0^ band is shifted toward lower values compared to the band in FePO_4_^11,44^.

The average spectrum belonging to the second group is presented in Fig. 4b. The spectrum is characterized by a very intense band centered around 1015 cm^-1^ and is correlated with symmetric stretching vibrations of P-O bonds in Q^0^ phosphate units. Its position is characteristic for α-FePO_4_^11,44^. Thus, the spectrum may be associated with the secondary rodolicoite phase.

### Ab initio calculations

The formation energy (E_form_) was calculated to check the thermodynamic stability of the proposed compound. The energy was calculated according to the following formula:

$${E}_{form}={E}_{0}-x{E}_{P}-y{E}_{Fe}-\frac{z}{2}{E}_{{O}_{2}}$$

where E_0_ is the total energy of the compound; E_P_, E_Fe_ are the energies of the stable P, Fe metals, while E_O2_ is the energy of the O_2_ molecule; x, y, z are the numbers of P, Fe, and O atoms in the unit cell, respectively.

The determined E_form_ energy is −2.316 eV/atom and its negative sign confirms the thermodynamic stability of the new Fe_7_P_11_O_38_ phase. The result is in accordance with the values of other iron-phosphate compounds summarized in the Materials Project database^45^, where the values are in range of about 2.0–2.5 eV/atom.

#### Magnetic properties

In the obtained phase, the magnetic moment may be governed by the existence of Fe^3+^ ions that have partially filled 3d orbitals. Three iron sites may be magnetically active. Two octahedral sites that are joined and form the [Fe_2_O_9_] dimers, and one tetrahedral [FeO_4_] that is separated from the dimers by [PO_4_] tetrahedrons.

The calculated iron magnetic moments in the dimers are coupled antiferromagnetically and are c.a. 4.144 µ_B_ and −4.104 µ_B_. The theoretical results are comparable to the experimental value of 4.55 µ_B_ for Fe_4_(P_2_O_7_)_3_^21^, where the symmetry and interatomic distances are similar, and also the magnetic moments are aligned antiferromagnetically. Similar results were also theoretically evidenced in analogous dimers in Fe_2_(HPO_3_)_3_^46^. It should be noted that the Fe–Fe distance in the dimers is relatively small c.a. 3.02 Å, and the Fe–O–Fe angle is close to 90°. This gives the possibility of predicting that the superexchange interaction is relatively small due to the angle that prefers a ferromagnetic alignment and that the direct interaction is dominant. This is supported by a detailed calculation of exchange integrals conducted by Kovrugin et al.^46^, where the superexchange interaction in the similar dimers prefers antiferromagnetic alignment, but the value is very small.

The interaction between [Fe_2_O_9_] groups is also antiferromagnetic. The exchange interactions occur along Fe–O–P–O–Fe paths via the super-superexchange mechanism. However, some magnetic frustration occurs because of the oxygen atoms of the shared faces. In this case, also some direct interaction may be influenced by the fact that the Fe–Fe intradimer distance is considerably shorter (4.78 Å) for the antiferromagnetically coupled iron moments than between the ferromagnetically coupled irons (6.05 Å). However, the Fe–Fe distance is relatively long and the Fe–O–P, P–O–P, and P–O–Fe angles in the Fe–O–P–O–Fe pathway vary in the range of 110°–167° and are far from 180°. Thus, we may expect that the d orbital overlap is poor and that the Neel temperature should be very low. Furthermore, PO_4_ groups are generally inefficient as a spin-exchange mediator^46^.

The more interesting problem is related to Fe^3+^ ions in tetrahedral coordination. In the case of the dimers, the values and alignment of the magnetic moments may be easily predicted by comparison to those of other iron phosphates of a similar local structure. The [FeO_4_] tetrahedrons are separated from the dimers by the [PO_4_] tetrahedrons, and the iron atoms cannot directly interact through the Fe–O–Fe superexchange interaction. Additionally, the direct Fe–Fe distances to iron atoms in the dimers are similar c.a. 4.77 Å and 4.98 Å. However, according to the calculations, a relatively weak magnetic moment of about 0.857 µ_B_ at the iron atoms is evidenced. That was probably due to super-superexchange interaction via the Fe–O–P–O–Fe pathway.

Finally, although the magnetic moments on the specific ions have non-integer values (Table S6—supplementary section), the calculated total magnetic moment of the unit cell has an integer value equal to −2.000 µ_B_/f.u. This phenomenon is relatively rare and it is characteristic for materials with half-metallic properties^47–49^.

The conducted calculations do not give a direct answer on the origin of magnetic properties of the phase and can be subject to more detailed theoretical and experimental studies.

#### Hyperfine interactions

The ab initio calculations give the unique possibility of estimating selected hyperfine interaction parameters that are directly measured by Mössbauer spectroscopy. One of the most frequently discussed parameters is isomer shift. The IS value is the result of changes in electron densities at the Mössbauer nucleus site (ρ(0)) and depends linearly on the densities. The line coefficients depend on the calculation method used and the exchange–correlation functional. More details can be found, e.g., in^36,50,51^. The calculated ρ(0) values are c.a. 15,308.009817 a.u./ Å^3^, and 15,307.834497 a.u./ Å^3^ for iron atoms in the dimers, and 15,309.226112 a.u./ Å^3^ for Fe^3+^ in the tetrahedral coordination. Taking into account the line coefficients summarized in^52^, obtained using the same calculation method, one may estimate the values of isomer shift at 0 K. Thus, the predicted IS values are 0.566 mm/s, 0.621 mm/s for iron in the dimers and 0.182 mm/s for coordinated tetrahedral. The measured values (Table 1) are in reasonable agreement with the theoretically predicted values, taking into account that the measurements were conducted at 300 K and the values need to be lower due to the second-order Doppler shift.

Similarly to the IS, a quadrupole split parameter (e^2^Q/2) should be proportional to an electronic field gradient (EFG). Calculated EFGs are following 2.533·10^21^ V/m^2^, 4.641·10^21^ V/m^2^, 4.426·10^21^ V/m^2^ for irons in the dimers and tetrahedral coordination, respectively.

Thus, we may expect that the theoretical Mössbauer spectrum of the phase is composed of three components. Two of the similar values of IS and different e^2^Q/2, whereas the lower one is about 55% of the higher, and are associated with the dimers. The third has a considerably lower value of IS, and the e^2^Q/2 value is slightly lower than the higher e^2^Q/2 component in the dimers. These agree very well with the experimental results of Mössbauer (see Table 1), where the lower e^2^Q/2 value is about 60% higher, and the tetrahedrally coordinated iron has only slightly lower e^2^Q/2 value.

It should also be pointed out that at an appropriate low temperature the spectrum should be magnetically split because of the occurrence of the predicted magnetic order. If we assume that, as in many oxides, the iron magnetic moment is proportional to the magnetic hyperfine field, one may estimate the values of the field. Taking the proportionality constant 13 T/µ_B_^52,53^, the estimated magnetic hyperfine fields are the following: 53.87 T, 53.35 T, 11.14 T for iron atoms in the dimers and the tetrahedrons, respectively.

#### Electronic properties

**Figure 5:**
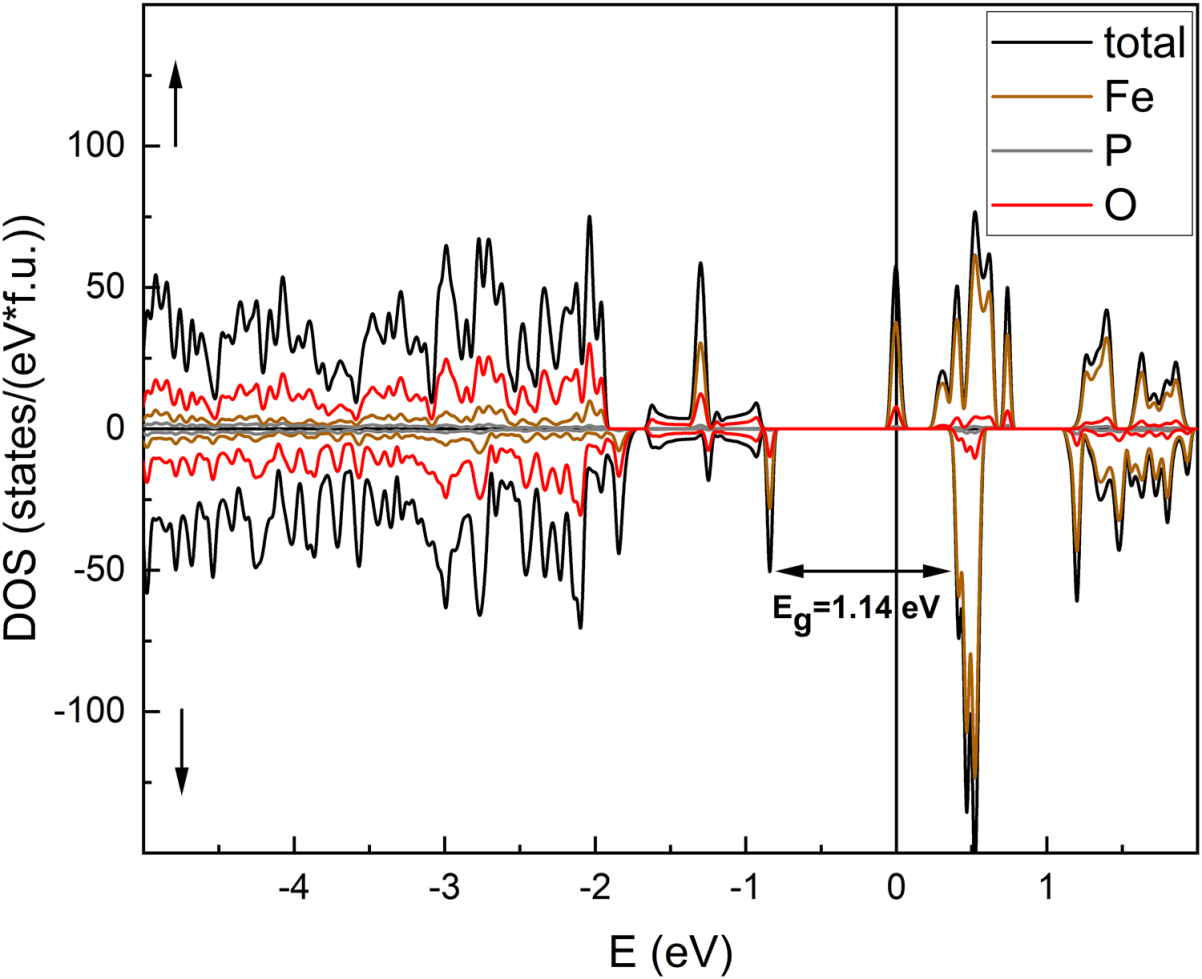
The total density of electronic states (DOS) of Fe_7_P_11_O_38_.

**Figure 6:**
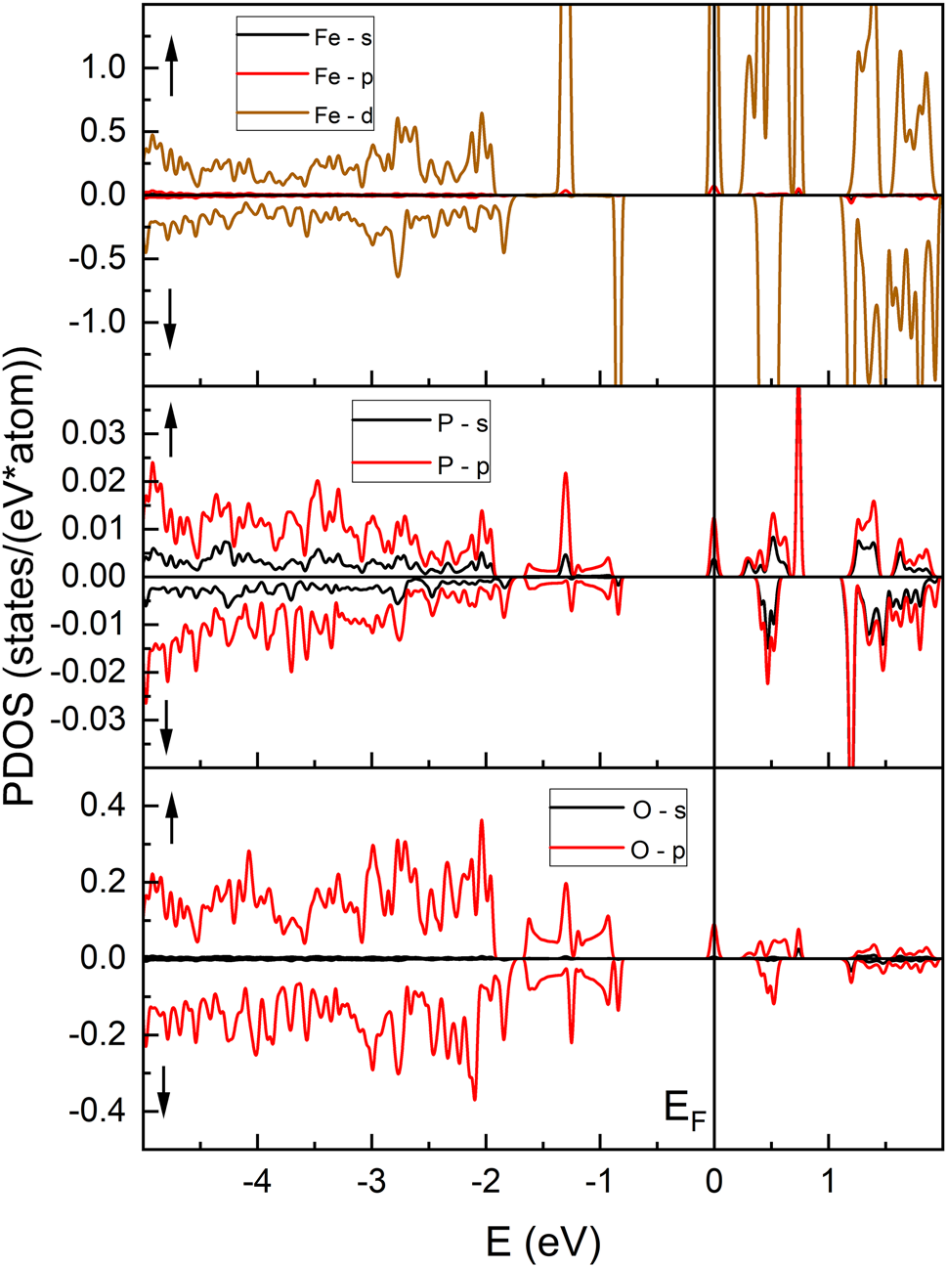
Partial densities of states (PDOS) for Fe_7_P_11_O_38_.

Electronic density of states (DOS) curves are widely used to investigate chemical bonding properties and to predict transport features of the material. The calculated total electronic densities (DOS) and partial (PDOS) of the states are presented in Figs. 5 and 6, respectively. The calculations were spin-resolved, and the majority spin band is designated as up.

It can be easily noticed that the spin up channel exhibits metallic behavior, whereas the down channel shows isolating features. This together with the integer value of the total magnetic moment gives the possibility of characterizing the phase as a half-metal. The spin-down valence and conduction bands are separated by the energy gap c.a. 1.14 eV. However, it should be noted that application of the U parameter in the calculations opens a band gap, and only comparison to experimental data may guarantee that the phase has half-metallic properties. The valence band is formed mainly by Fe 3d-states and O 2p-states with a little contribution of P s,p-states. However, it should be noted that, especially in the upper part of the valence band, some characteristic features of the shape of the band are common for O-2p, P-s,p, and Fe-3d, indicating the hybridization of the electrons, and may suggest the covalent character of P–O, Fe–O bonds. In the case of the spin-up channel at the E_F_ level, there is evidence of a partially filled conduction band. The band is narrow and is mostly created by the Fe 3d state. However, the s,p states of P and O both participate in the band formation.

**Figure 7:**
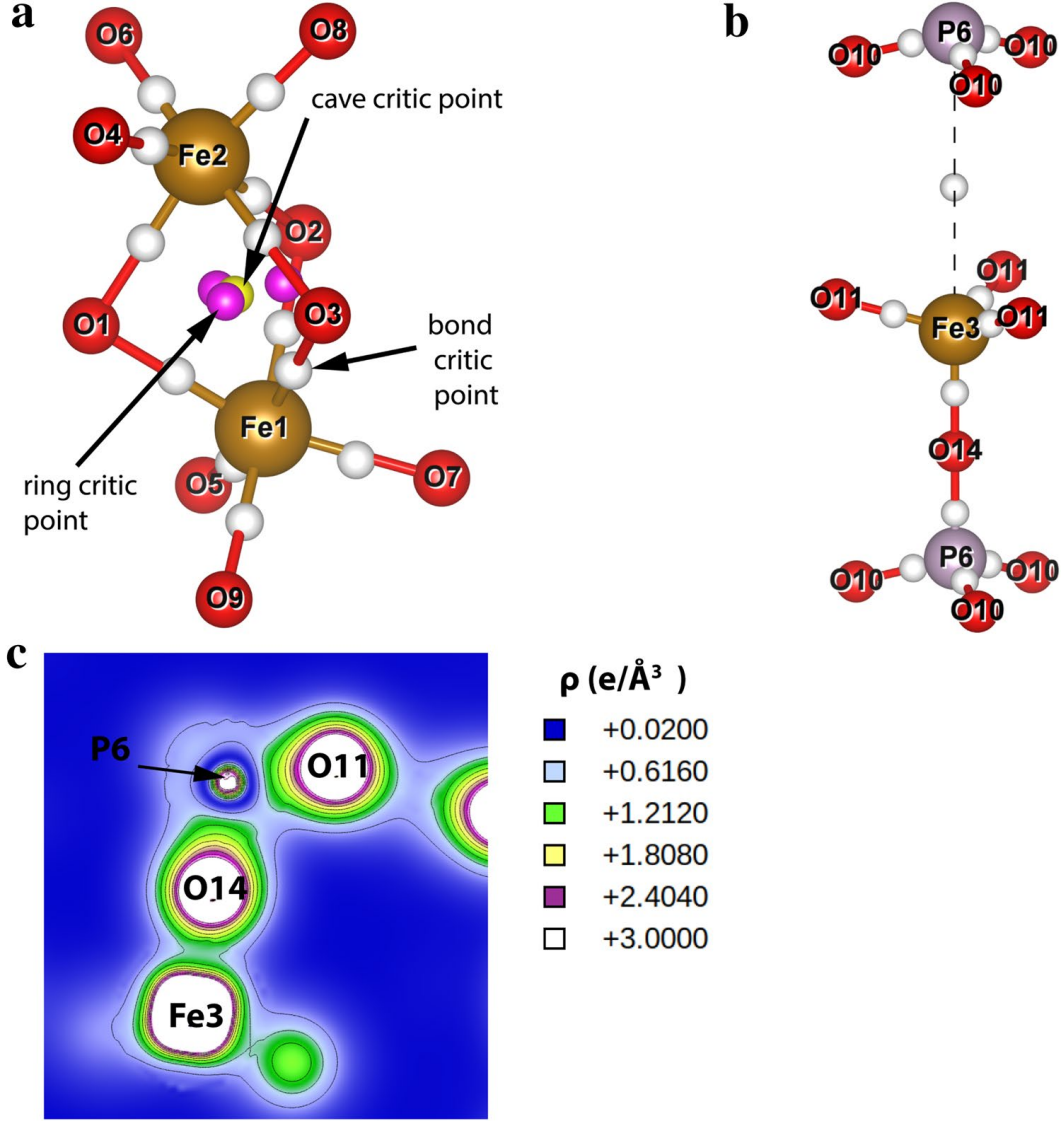
Critic points for **(a)** [Fe1O_6_]–[Fe2O_6_], **(b)** for [Fe3O_4_]–[PO_4_] and **(c)** valence electrons density map showing the connection Fe3–O14–P6 for Fe_7_P_11_O_38_.

The topological analysis according to the Bader formalism was performed^54,55^. The most important critical points and their properties are available in the supplementary material in Table S7. The selected chemical bonds containing these critical points and the corresponding valence electron density map for Fe3–O14–P6 are shown in Fig. 7. All critical points of the bond in the system have positive values of electron density Laplacian (${\nabla }^{2}\rho$) as well as electrostatic potential to the kinematic energy ratio $\frac{\left|V\right|}{G}$ lower than 2. This indicates that there are no purely covalent shared shell bonds in the system^56–58^. Values of $\frac{\left|V\right|}{G}$ ratio are between 1 and 2 for Fe–O-bond critic points both in iron octahedra and tetrahedra. These bonds have a transient ion-covalent character. Bond critic points in tetrahedra have little bigger $\frac{\left|V\right|}{G}$ values than bond critic points in octahedra. This shows a more covalent character of the bonds in iron tetrahedra. The octahedra for Fe1 and Fe2 are connected by face (Fig. 7. a), between Fe1 and Fe2 is cave critic point with a relatively high value of $\rho$ = 0.027 $\frac{e}{{\AA }^{3}}$ and $\frac{\left|V\right|}{G}$  = 1.156 as well as ring critic points in [Fe1O_6_]-[Fe2O_6_] fragment which have $\rho >0.01 \frac{e}{{\AA }^{3}}$ and $\frac{\left|V\right|}{G}$ > 1. Other points of ring and cave critic points in system have $\rho \approx {10}^{-3}\frac{e}{{\AA }^{3}}$ and $\frac{\left|V\right|}{G}<1$. This may suggest some interaction between these irons. Bond critic points for P-O have higher values of $\rho$ and $\frac{\left|V\right|}{G}$ than bond critic points for Fe–O and more covalent character. Figure 7c shows the valence electron density map for the Fe3-O14-P6 connection. This map clearly shows that the regions between Fe3 and O14 or P4 and O14 with an electron density about 0.1 $e/{\AA }^{3}$. A more detailed view shows that the bond critic point for P6-O14 has higher values of $\rho$ and $\frac{\left|V\right|}{G}$, than bond critic point for P6–O14. This shows a more covalent character of the P6-O14 bond. The bond critic point between Fe3 and P6 (Fig. 7b) have $\rho \approx {10}^{-3} e/{\AA }^{3} \mathrm{and} \frac{\left|V\right|}{G}< 1$. This indicates a pure closed-shell interaction such as an ionic interaction. Because Fe3 and P5 share O14 the formal charge inside the phosphorus tetrahedra is positive, while in the iron tetrahedra it is negative.

It can be clearly seen (Fig. 7c) that in the middle between the Fe–O and P–O atoms there is no vanishing valence electron density that exhibits a directional distribution. This confirms the partially covalent character of P–O and Fe–O bonds.

## Conclusions

The new Fe_7_P_11_O_38_ compound was detected as the result of the glass devitrification of 40Fe_2_O_3_–60P_2_O_5_. The compound crystallizes in the hexagonal P6_3_ space group and crystal structure parameters were obtained.

The ^57^Fe Mössbauer effect measurements of the compound were conducted and the hyperfine interaction parameters were determined. The results confirmed the octahedral and tetrahedral positions of iron in the crystal structure.

Raman spectroscopy measurements were conducted. The newly developed phase vibrations were observed and appropriate assignments were made.

The basic magnetic and electric features of the compound were predicted using ab initio simulations. It was observed that the iron magnetic moments are coupled antiferromagnetically in and between [Fe_2_O_9_] dimers. The total magnetic moment of the unit cell has a unique integer value. The calculated electronic density of states revealed that a majority spin band has metallic character, whereas the minority is isolating. Thus, the compound may be designated as a half-metal.

The bond critical point analysis showed the ionic-covalent nature of P–O and Fe–O bonds. The P–O bonds are more covalent than Fe–O. The tetrahedrally coordinated iron has a covalent character more than that of the octahedrally coordinated iron.

## Materials and methods

Pyrophosphate stoichiometry glass of the general formulae 40Fe_2_O_3_–60P_2_O_5_ (mol%) was synthesized using a conventional glass melting and quenching technique. The batch was prepared by careful homogenization in a planetary ball mill with appropriate amounts of NH_4_H_2_PO_4_ and Fe_2_O_3_ with high chemical purity. The mixture was melted in an electric laboratory furnace in Al_2_O_3_ crucibles. The melting temperature was 1473 K. The melt was vitrified by casting it onto a steel plate. During melting at temperatures above 1300 K, there was evidence of evaporation of P_2_O_5_^59^. To compensate for P_2_O_5_ losses, an approximately 20% overweight of NH_4_H_2_PO_4_ was used. The chemical composition of the obtained glass was verified by X-ray fluorescence spectroscopy (XRF) and was consistent with the assumption in the experimental uncertainty limit. A small, approximately 1 mol% overweight of P_2_O_5_ was detected over the assumed stoichiometry and below 1 mol% addition of Al_2_O_3_. The amorphous nature of the investigated materials was confirmed by X-ray diffraction (XRD). The obtained XRD pattern was fully amorphous with a single broad halo with a maximum of around 2Θ≈20°. Crystalline peaks were not detected. The glass was milled and the powder was devitrified in a laboratory electric furnace for 48 h in an air atmosphere at a temperature of 1140 K. The glass powder was not pressed prior to the crystallization step, but was poured directly into the ceramic alumina combustion boat. Therefore, the sample after crystallization has an irregular form, which disintegrated into a powder under low force. The sample after crystallization was dull gray in color.

The sample for XRF was performed by pressing glass powders into thin tablets. The investigation was carried out using an Axios mAX WDXRF X-ray fluorescence spectrometer with an Rh lamp of 4 kW power (PANalytical). The analysis was carried out using the standardless method. The uncertainty of measurement was about 5%.

Powder XRD measurements of glass and devitrified materials were carried out with a Philips X’Pert Pro diffractometer and Cu Kα1 radiation. The phase compositions of the crystallized samples and the crystal structure parameters have been obtained by the Rietveld method using GSAS-II software^60^. Structural parameters, including scale factor, zero shift, background function, lattice parameters, atomic coordinates, and peak profile, were taken into account in the course of refinement. To determine the space group of the unknown phase, the EXPO2014 software was used^29^.

Mössbauer transmission measurements were performed using an MsAa-3 spectrometer (RENON, Kraków, Poland)^61^ equipped with an LND Kr-filled proportional detector and a He–Ne laser-based interferometer. A single-line commercial ^57^Co(Rh) source kept at room temperature was applied for a 14.41 keV resonant transition in ^57^Fe. The Mössbauer absorbers were prepared in powder form by mixing 80 mg of the investigated material with a B_4_C carrier and lightly pressing in a sample holder between biaxially oriented polyethylene terephthalate window sheets aluminized on both sides. Therefore, the absorber thickness was approximately 40 mg cm^–2^ of the investigated material, since the circular sample holder has a diameter of 16 mm. Spectra were collected for absorbers kept at room temperature. The obtained spectra were least-square fitted using full static site Hamiltonian analysis^62^.

All Raman measurements were made using a Witec alpha 300 M + Confocal Raman Imaging system with the application of a 50 × air objective (Zeiss, LD EC Epiplan-Neofluar, NA = 0.55). The spectrometer was equipped with an air-cooled solid-state laser operating at 488 nm, a CCD detector that was cooled to −60 °C, and 600 grooves per mm of gratings. Ten randomly chosen Raman spectra of each glass powder sample were collected with 2 scans and an integration time of 20 s. Raman spectra were normalized and then deconvoluted using PeakFit software. The Gaussian–Lorentzian bands' shapes were used in the deconvolution process. During the deconvolution procedure, no constraints were used; e.g., intensity, width, and position of the fitted peaks can change freely. The error in the observed deconvolution was less than 1%.

Electronic band structure calculations were performed using the ab initio self-consistent full potential linearized augmented plane waves (FLAPW) method implemented in the WIEN2k code^63^. The generalized gradient approximation (GGA) was used in the parameterization of Perdew–Burke–Ernzerhof revised for solids (PBEsol)^64^. Because it is well-known that GGA methods underestimate the value of the energy gap in strongly localized d-electrons, the electronic properties have been calculated using the GGA + U method. For the calculations, the Hubbard parameter U was set to 5 eV with J = 0 for all iron atoms. The value of the U parameter was chosen based on reports from the previous literature about iron in oxides and phosphates^65–67^. Brillouin zone integration was performed using a k mesh of 162 k points in the irreducible Brillouin zone wedge. The plane-wave cutoff for the basis function was set to RK_max_ = 7.0. The crystal structure parameters were set according to the XRD results. All calculations were conducted as the spin resolved. The rest of the parameters were set as default as implemented in the Wien2k code. The electronic density of states was calculated with the application of Gaussian smearing with the width of 13 meV.

Topological analysis of total electron densities was carried out according to Bader’s formalism employing the CRITIC2 program^68^.

## Supplementary Information

Supplementary Information.

Supplementary Tables.
