# Supplementary material for: A new iron-phosphate compound (Fe7P11O38) obtained by pyrophosphate stoichiometric glass devitrification
Source: Sci Rep. 2021 Nov 25;11:22957. doi: 10.1038/s41598-021-02471-0 (PMC8617057; doi:10.1038/s41598-021-02471-0)
Supplement: Supplementary file 2 — Supplementary Tables. [file 41598_2021_2471_MOESM2_ESM.docx]

Table S1. Crystal data for new Fe_7_P_11_O_38_ phase.

| Space Group | P6_3_ |
| --- | --- |
| Cell dimensions | |
| a, b, c (Å) | 14.4504(16), 14.4504(16), 7.4261(2) |
| $\alpha, \beta, \gamma$ (^o^) | 90, 90, 120 |

Table S2. Atomic coordinates for new Fe_7_P_11_O_38_ phase.

| Position | x | y | z | site symmetry multiplicity |
| --- | --- | --- | --- | --- |
| Fe1 | 0.343(4) | 0.321(5) | 0.0316(23) | 6 |
| Fe2 | 0.338(4) | 0.322(4) | 0.4380(20) | 6 |
| P1 | 0.2181(25) | 0.4396(29) | 0.227(10) | 6 |
| P2 | 0.5130(26) | 0.0890(26) | 0.247(9) | 6 |
| P3 | 0.2318(27) | 0.0910(29) | 0.234(10) | 6 |
| P4 | 0 | 0 | 0.260(13) | 2 |
| Fe3 | 0.66667 | 0.33333 | 0.206(19) | 2 |
| P6 | 0.33333 | 0.66667 | 0.262(15) | 2 |
| O1 | 0.303(5) | 0.204(5) | 0.237(22) | 6 |
| O2 | 0.464(5) | 0.408(5) | 0.230(22) | 6 |
| O3 | 0.270(5) | 0.371(6) | 0.242(23) | 6 |
| O4 | 0.254(9) | 0.045(10) | 0.075(14) | 6 |
| O5 | 0.240(8) | 0.037(9) | 0.419(15) | 6 |
| O6 | 0.266(7) | 0.854(8) | 0.060(14) | 6 |
| O7 | 0.278(8) | 0.851(9) | 0.380(13) | 6 |
| O8 | 0.448(11) | 0.058(11) | 0.080(15) | 6 |
| O9 | 0.456(9) | 0.054(9) | 0.426(16) | 6 |
| O10 | 0.249(5) | 0.694(6) | 0.204(13) | 6 |
| O11 | 0.565(5) | 0.209(5) | 0.270(16) | 6 |
| O12 | 0 | 0 | 0.975(21) | 2 |
| O13 | 0.117(5) | 0.076(6) | 0.196(13) | 6 |
| O14 | 0.66667 | 0.33333 | -0.030(19) | 2 |

Table S3. Interatomic distances of i-j atoms for new Fe_7_P_11_O_38_ phase.

| Atom i site | Atom j site | Distance (Å) |
| --- | --- | --- |
| FeO6 -octahedra | | |
| Fe1 | O1 | 2.13(13) |
| Fe1 | O2 | 2.14(13) |
| Fe1 | O3 | 2.20(14) |
| Fe1 | O5 | 1.95(12) |
| Fe1 | O7 | 1.98(11) |
| Fe1 | O9 | 1.86(14) |
| Fe2 | O1 | 2.13(14) |
| Fe2 | O2 | 2.23(12) |
| Fe2 | O3 | 2.07(13) |
| Fe2 | O4 | 1.90(11) |
| Fe2 | O6 | 1.87(11) |
| Fe2 | O8 | 1.90(14) |
| FeO4 -tetrahedra | | |
| Fe3 | O11 | 1.73(5) |
| Fe3 | O11 | 1.73(5) |
| Fe3 | O11 | 1.73(5) |
| Fe3 | O14 | 1.76(10) |
| PO4-terahedra | | |
| P1 | O3 | 1.51(6) |
| P1 | O6 | 1.54(10) |
| P1 | O7 | 1.46(10) |
| P1 | O10 | 1.52(6) |
| P2 | O2 | 1.45(7) |
| P2 | O8 | 1.48(10) |
| P2 | O9 | 1.51(10) |
| P2 | O11 | 1.51(6) |
| P3 | O1 | 1.44(7) |
| P3 | O4 | 1.47(10) |
| P3 | O5 | 1.61(11) |
| P3 | O13 | 1.59(6) |
| P4 | O12 | 1.60(12) |
| P4 | O13 | 1.56(6) |
| P4 | O13 | 1.56(6) |
| P4 | O13 | 1.56(6) |
| P6 | O10 | 1.52(5) |
| P6 | O10 | 1.52(5) |
| P6 | O10 | 1.52(5) |
| P6 | O14 | 1.54(10) |

Table S4. Interatomic angles of i-j-k atoms for new Fe_7_P_11_O_38_ phase.

| Atom i site | Atom j site | Atom k site | Angle (^o^) |
| --- | --- | --- | --- |
| FeO6 -octahedra | | | |
| O1 | Fe1 | O2 | 78(5) |
| O1 | Fe1 | O3 | 77(5) |
| O2 | Fe1 | O3 | 73(5) |
| O1 | Fe1 | O5 | 92(4) |
| O2 | Fe1 | O5 | 161(5) |
| O3 | Fe1 | O5 | 89(4) |
| O1 | Fe1 | O7 | 97(5) |
| O2 | Fe1 | O7 | 97(4) |
| O3 | Fe1 | O7 | 169(6) |
| O5 | Fe1 | O7 | 101(5) |
| O1 | Fe1 | O9 | 159(6) |
| O2 | Fe1 | O9 | 82(6) |
| O3 | Fe1 | O9 | 90(5) |
| O5 | Fe1 | O9 | 104(6) |
| O7 | Fe1 | O9 | 93(5) |
| O1 | Fe2 | O2 | 76(4) |
| O1 | Fe2 | O3 | 80(5) |
| O2 | Fe2 | O3 | 74(4) |
| O1 | Fe2 | O4 | 101(4) |
| O2 | Fe2 | O4 | 166(5) |
| O3 | Fe2 | O4 | 93(5) |
| O1 | Fe2 | O6 | 87(4) |
| O2 | Fe2 | O6 | 95(4) |
| O3 | Fe2 | O6 | 164(6) |
| O4 | Fe2 | O6 | 98(5) |
| O1 | Fe2 | O8 | 168(6) |
| O2 | Fe2 | O8 | 92(6) |
| O3 | Fe2 | O8 | 96(5) |
| O4 | Fe2 | O8 | 91(5) |
| O6 | Fe2 | O8 | 96(5) |
| FeO4 -tetrahedra | | | |
| O10 | P6 | O10 | 112.4(29) |
| O10 | P6 | O10 | 112.4(29) |
| O10 | P6 | O10 | 112.4(29) |
| O10 | P6 | O14 | 106(3) |
| O10 | P6 | O14 | 106(3) |
| O10 | P6 | O14 | 106(3) |
| PO4-terahedra | | | |
| O3 | P1_P | O6 | 112(4) |
| O3 | P1_P | O7 | 113(4) |
| O6 | P1_P | O7 | 105(3) |
| O3 | P1_P | O10 | 108(4) |
| O6 | P1_P | O10 | 106(4) |
| O7 | P1_P | O10 | 111(4) |
| O2 | P2_P | O8 | 108(4) |
| O2 | P2_P | O9 | 111(4) |
| O8 | P2_P | O9 | 118(4) |
| O2 | P2_P | O11 | 111(3) |
| O8 | P2_P | O11 | 109(4) |
| O9 | P2_P | O11 | 99(4) |
| O1 | P3_P | O4 | 110(4) |
| O1 | P3_P | O5 | 111(4) |
| O4 | P3_P | O5 | 113(3) |
| O1 | P3_P | O13 | 105(4) |
| O4 | P3_P | O13 | 104(4) |
| O5 | P3_P | O13 | 114(4) |
| O12 | P4_P | O13 | 107.9(22) |
| O12 | P4_P | O13 | 107.9(22) |
| O13 | P4_P | O13 | 111.0(20) |
| O12 | P4_P | O13 | 107.9(22) |
| O13 | P4_P | O13 | 111.0(20) |
| O13 | P4_P | O13 | 111.0(20) |
| O10 | P6_P | O10 | 112.4(29) |
| O10 | P6_P | O10 | 112.4(29) |
| O10 | P6_P | O10 | 112.4(29) |
| O10 | P6_P | O14 | 106(3) |
| O10 | P6_P | O14 | 106(3) |
| O10 | P6_P | O14 | 106(3) |

Table S5. Intensity normalized to 100 I_100_, observed F_o_ and calculated F_c_ structure-factor moduli and d_hkl_-spacing for new Fe_7_P_11_O_38_ phase.

| (h k l) | 2-theta | I_100_ | F_o_ | F_c_ | d_hkl_-spacing (Å) |
| --- | --- | --- | --- | --- | --- |
| (1 1 0) | 12.31313 | 18.560 | 87.88921 | 62.99932 | 7.22521 |
| (0 1 1) | 13.92816 | 0.977 | 16.39326 | 8.523556 | 6.38632 |
| (0 2 0) | 14.21546 | 0.811 | 21.40315 | 7.413029 | 6.25721 |
| (1 1 1) | 17.18102 | 8.257 | 58.21025 | 38.42554 | 5.17855 |
| (0 2 1) | 18.59967 | 0.912 | 20.92097 | 17.17504 | 4.78505 |
| (1 2 0) | 18.81714 | 0.898 | 29.68036 | 22.71438 | 4.73001 |
| (2 1 0) | 18.81714 | 0.104 | 10.08266 | 7.716281 | 4.73001 |
| (0 3 0) | 21.35403 | 55.520 | 264.2258 | 252.811 | 4.17148 |
| (1 2 1) | 22.3369 | 0.263 | 26.18152 | 22.05049 | 3.98947 |
| (2 1 1) | 22.3369 | 0.986 | 13.52342 | 11.38964 | 3.98947 |
| (0 0 2) | 24.01792 | 5.913 | 168.497 | 165.2407 | 3.71304 |
| (0 3 1) | 24.52649 | 100.000 | 288.6457 | 284.6416 | 3.63695 |
| (2 2 0) | 24.69382 | 0.085 | 12.06039 | 12.99057 | 3.6126 |
| (0 1 2) | 25.06584 | 0.470 | 20.7856 | 20.25991 | 3.55966 |
| (1 3 0) | 25.71581 | 1.759 | 37.85271 | 35.89522 | 3.47088 |
| (3 1 0) | 25.71581 | 0.680 | 60.88301 | 57.73456 | 3.47088 |
| (1 1 2) | 27.04731 | 33.528 | 184.9398 | 182.5782 | 3.30248 |
| (2 2 1) | 27.50333 | 15.997 | 129.8999 | 126.9605 | 3.24859 |
| (0 2 2) | 27.98902 | 0.196 | 14.6522 | 11.57592 | 3.19316 |
| (1 3 1) | 28.43106 | 0.910 | 31.98762 | 30.49479 | 3.14438 |
| (3 1 1) | 28.43106 | 1.902 | 46.63255 | 44.45626 | 3.14438 |
| (0 4 0) | 28.57701 | 2.205 | 71.20492 | 52.97407 | 3.12861 |
| (1 2 2) | 30.65415 | 4.225 | 74.83666 | 56.56614 | 2.92065 |
| (2 1 2) | 30.65415 | 3.299 | 65.97449 | 49.86756 | 2.92065 |
| (0 4 1) | 31.06143 | 1.225 | 40.64922 | 36.7641 | 2.88318 |
| (2 3 0) | 31.19613 | 0.890 | 49.20446 | 45.14479 | 2.87101 |
| (3 2 0) | 31.19613 | 1.847 | 70.91663 | 65.06556 | 2.87101 |
| (0 3 2) | 32.31959 | 32.504 | 217.5859 | 214.0425 | 2.77349 |
| (1 4 0) | 32.8368 | 8.166 | 120.5238 | 116.9527 | 2.73087 |
| (4 1 0) | 32.8368 | 4.828 | 156.7395 | 152.0953 | 2.73087 |
| (2 3 1) | 33.50435 | 0.930 | 38.24873 | 33.08955 | 2.67785 |
| (3 2 1) | 33.50435 | 0.572 | 29.95875 | 25.91777 | 2.67785 |
| (2 2 2) | 34.68322 | 17.973 | 173.7871 | 165.3825 | 2.58928 |
| (4 1 1) | 35.04852 | 8.627 | 121.7123 | 115.3487 | 2.56306 |
| (1 4 1) | 35.04852 | 0.151 | 16.08263 | 15.24175 | 2.56306 |
| (1 3 2) | 35.44009 | 0.570 | 31.67554 | 30.0624 | 2.53557 |
| (3 1 2) | 35.44009 | 0.220 | 19.57963 | 18.58249 | 2.53557 |
| (0 5 0) | 35.91747 | 0.289 | 32.31459 | 24.73564 | 2.50289 |
| (0 1 3) | 37.05736 | 0.026 | 6.842295 | 8.504646 | 2.42831 |
| (3 3 0) | 37.37424 | 23.003 | 299.8773 | 296.1465 | 2.4084 |
| (0 4 2) | 37.63102 | 0.214 | 20.54171 | 15.46186 | 2.39252 |
| (0 5 1) | 37.97166 | 0.134 | 16.39613 | 20.237 | 2.37179 |
| (2 4 0) | 38.08463 | 0.075 | 17.43198 | 18.85959 | 2.365 |
| (4 2 0) | 38.08463 | 0.470 | 43.59774 | 47.16823 | 2.365 |
| (1 1 3) | 38.47695 | 0.825 | 41.36815 | 35.70028 | 2.34174 |
| (0 2 3) | 39.1703 | 0.302 | 25.94259 | 17.98597 | 2.30179 |
| (3 3 1) | 39.36312 | 2.481 | 73.39605 | 68.61423 | 2.29093 |
| (2 3 2) | 39.71795 | 0.143 | 17.61377 | 11.98703 | 2.27124 |
| (3 2 2) | 39.71795 | 0.628 | 37.33891 | 25.41096 | 2.27124 |
| (2 4 1) | 40.04353 | 1.132 | 14.34211 | 12.37477 | 2.25348 |
| (4 2 1) | 40.04353 | 0.092 | 50.48467 | 43.55964 | 2.25348 |
| (5 1 0) | 40.15157 | 0.468 | 46.0142 | 32.22304 | 2.24766 |
| (1 5 0) | 40.15157 | 0.045 | 14.2803 | 10.0003 | 2.24766 |
| (4 1 2) | 41.05951 | 6.677 | 125.623 | 112.0409 | 2.19993 |
| (1 4 2) | 41.05951 | 0.821 | 44.13845 | 39.36632 | 2.19993 |
| (1 2 3) | 41.19135 | 0.396 | 30.82632 | 22.60613 | 2.19318 |
| (2 1 3) | 41.19135 | 0.070 | 13.00458 | 9.536771 | 2.19318 |
| (1 5 1) | 42.02957 | 0.353 | 29.39379 | 21.76307 | 2.15128 |
| (5 1 1) | 42.02957 | 0.147 | 19.11243 | 14.1508 | 2.15128 |
| (0 3 3) | 42.49434 | 6.000 | 123.3694 | 109.6214 | 2.12877 |
| (0 6 0) | 43.41327 | 0.775 | 64.10932 | 59.86332 | 2.08574 |
| (0 5 2) | 43.64018 | 0.521 | 37.32054 | 33.98817 | 2.0754 |
| (3 4 0) | 44.04194 | 0.468 | 50.59292 | 44.99506 | 2.05736 |
| (4 3 0) | 44.04194 | 1.811 | 99.3246 | 88.3348 | 2.05736 |
| (2 2 3) | 44.39033 | 8.036 | 149.1734 | 137.4261 | 2.04199 |
| (3 3 2) | 44.88539 | 0.442 | 35.21356 | 31.61019 | 2.02057 |
| (1 3 3) | 45.0081 | 0.361 | 32.1349 | 27.173 | 2.01534 |
| (3 1 3) | 45.0081 | 0.625 | 42.17564 | 35.66336 | 2.01534 |
| (0 6 1) | 45.18036 | 0.817 | 48.46119 | 44.01274 | 2.00804 |
| (2 5 0) | 45.27835 | 0.251 | 61.33806 | 61.10429 | 2.00391 |
| (5 2 0) | 45.27835 | 0.653 | 38.03752 | 37.89256 | 2.00391 |
| (2 4 2) | 45.49785 | 1.593 | 47.36942 | 44.15244 | 1.99474 |
| (4 2 2) | 45.49785 | 0.773 | 68.12715 | 63.50046 | 1.99474 |
| (3 4 1) | 45.78972 | 0.980 | 53.70845 | 35.9413 | 1.98268 |
| (4 3 1) | 45.78972 | 0.161 | 21.87366 | 14.63769 | 1.98268 |
| (0 4 3) | 46.82285 | 1.690 | 72.14997 | 50.85522 | 1.94123 |
| (2 5 1) | 46.98991 | 0.094 | 17.14013 | 10.44955 | 1.93471 |
| (5 2 1) | 46.98991 | 0.431 | 36.75232 | 22.40618 | 1.93471 |
| (1 5 2) | 47.29794 | 1.252 | 62.67648 | 44.3741 | 1.9228 |
| (5 1 2) | 47.29794 | 1.889 | 77.07127 | 54.56541 | 1.9228 |
| (1 6 0) | 47.67539 | 0.013 | 88.33424 | 63.9687 | 1.90843 |
| (6 1 0) | 47.67539 | 1.222 | 9.013601 | 6.527327 | 1.90843 |
| (2 3 3) | 48.58503 | 0.390 | 35.79823 | 30.3061 | 1.87475 |
| (3 2 3) | 48.58503 | 1.652 | 74.15795 | 62.78073 | 1.87475 |
| (0 0 4) | 49.09245 | 6.651 | 0.189737 | 1 | 1.85652 |
| (1 6 1) | 49.32282 | 0.442 | 10.31513 | 9.987042 | 1.84837 |
| (6 1 1) | 49.32282 | 0.031 | 38.78006 | 37.54668 | 1.84837 |
| (0 1 4) | 49.6648 | 0.081 | 16.88434 | 16.15144 | 1.83642 |
| (1 4 3) | 49.73355 | 9.529 | 181.9202 | 177.1617 | 1.83404 |
| (4 1 3) | 49.73355 | 7.079 | 156.856 | 152.7531 | 1.83404 |
| (0 6 2) | 50.18776 | 9.670 | 184.9721 | 181.9989 | 1.81847 |
| (4 4 0) | 50.54898 | 1.875 | 116.0951 | 117.6616 | 1.8063 |
| (3 4 2) | 50.75099 | 0.070 | 15.71572 | 12.07974 | 1.79957 |
| (4 3 2) | 50.75099 | 0.148 | 22.87219 | 17.5805 | 1.79957 |
| (1 1 4) | 50.7952 | 0.072 | 16.35897 | 11.6245 | 1.79811 |
| (0 7 0) | 51.10931 | 0.005 | 6.29182 | 4.58988 | 1.78778 |
| (3 5 0) | 51.10931 | 1.034 | 72.17236 | 52.65028 | 1.78778 |
| (5 3 0) | 51.10931 | 0.713 | 87.18363 | 63.60112 | 1.78778 |
| (0 2 4) | 51.35358 | 0.421 | 39.50167 | 27.82959 | 1.77983 |
| (2 5 2) | 51.86411 | 0.119 | 21.11319 | 19.88899 | 1.76348 |
| (5 2 2) | 51.86411 | 7.036 | 162.9505 | 153.5022 | 1.76348 |
| (0 5 3) | 51.97421 | 1.042 | 62.82722 | 62.86932 | 1.75999 |
| (4 4 1) | 52.12888 | 1.302 | 70.52383 | 60.52599 | 1.75513 |
| (0 7 1) | 52.67711 | 0.287 | 33.45826 | 26.57043 | 1.73812 |
| (3 5 1) | 52.67711 | 0.349 | 36.75187 | 29.18601 | 1.73812 |
| (5 3 1) | 52.67711 | 0.052 | 14.28394 | 11.34341 | 1.73812 |
| (2 6 0) | 52.76451 | 0.083 | 33.47778 | 30.01768 | 1.73544 |
| (6 2 0) | 52.76451 | 0.144 | 25.39896 | 22.77384 | 1.73544 |
| (1 2 4) | 53.00333 | 0.135 | 23.56828 | 18.80165 | 1.72817 |
| (2 1 4) | 53.00333 | 0.203 | 28.23324 | 22.52314 | 1.72817 |
| (3 3 3) | 53.06894 | 0.243 | 30.79385 | 21.64311 | 1.72618 |
| (2 4 3) | 53.61041 | 2.351 | 97.28522 | 72.48816 | 1.70999 |
| (4 2 3) | 53.61041 | 0.079 | 18.05381 | 13.45206 | 1.70999 |
| (1 6 2) | 54.04113 | 0.380 | 39.4315 | 34.61358 | 1.69736 |
| (6 1 2) | 54.04113 | 2.568 | 102.4773 | 89.95618 | 1.69736 |
| (0 3 4) | 54.08342 | 0.496 | 45.06929 | 42.21065 | 1.69613 |
| (2 6 1) | 54.29844 | 0.320 | 44.43818 | 31.24002 | 1.68991 |
| (6 2 1) | 54.29844 | 0.480 | 36.3696 | 25.56781 | 1.68991 |
| (1 5 3) | 55.21276 | 0.222 | 30.51452 | 27.21253 | 1.66402 |
| (5 1 3) | 55.21276 | 1.109 | 68.82425 | 61.37673 | 1.66402 |
| (7 1 0) | 55.44559 | 0.496 | 65.3773 | 53.41744 | 1.65758 |
| (1 7 0) | 55.44559 | 0.307 | 51.42877 | 42.02059 | 1.65758 |
| (2 2 4) | 55.67648 | 0.644 | 52.8369 | 46.67726 | 1.65124 |
| (1 3 4) | 56.20073 | 0.329 | 38.16432 | 45.11484 | 1.63705 |
| (3 1 4) | 56.20073 | 0.032 | 11.73473 | 13.87188 | 1.63705 |
| (4 4 2) | 56.68083 | 0.212 | 30.85549 | 25.74057 | 1.6243 |
| (1 7 1) | 56.9301 | 2.803 | 37.98333 | 40.42679 | 1.61777 |
| (7 1 1) | 56.9301 | 0.320 | 112.6174 | 119.862 | 1.61777 |
| (0 7 2) | 57.19903 | 0.500 | 47.7869 | 42.39708 | 1.61079 |
| (3 5 2) | 57.19903 | 0.042 | 13.83456 | 12.27416 | 1.61079 |
| (5 3 2) | 57.19903 | 0.171 | 28.05582 | 24.89144 | 1.61079 |
| (4 5 0) | 57.52929 | 2.374 | 147.996 | 147.2901 | 1.60231 |
| (5 4 0) | 57.52929 | 0.023 | 14.48713 | 14.41804 | 1.60231 |
| (0 4 4) | 57.7547 | 0.121 | 23.69694 | 25.8456 | 1.59658 |
| (0 6 3) | 57.81664 | 2.323 | 103.9766 | 102.815 | 1.59502 |
| (3 4 3) | 58.32833 | 1.679 | 89.12892 | 81.18022 | 1.58222 |
| (4 3 3) | 58.32833 | 0.739 | 59.18327 | 53.90519 | 1.58222 |
| (6 3 0) | 58.55303 | 8.928 | 291.8902 | 283.7356 | 1.57667 |
| (3 6 0) | 58.55303 | 2.471 | 153.6328 | 149.3407 | 1.57667 |
| (2 6 2) | 58.73591 | 0.857 | 64.18663 | 57.35469 | 1.57219 |
| (6 2 2) | 58.73591 | 0.360 | 41.35015 | 36.9489 | 1.57219 |
| (5 4 1) | 58.9796 | 0.098 | 21.83598 | 17.5038 | 1.56626 |
| (4 5 1) | 58.9796 | 0.057 | 16.74055 | 13.41928 | 1.56626 |
| (0 8 0) | 59.06069 | 0.154 | 38.62355 | 34.87787 | 1.5643 |
| (2 3 4) | 59.28242 | 0.006 | 5.262604 | 5.322687 | 1.55897 |
| (3 2 4) | 59.28242 | 0.573 | 52.80738 | 53.41001 | 1.55897 |
| (2 5 3) | 59.34336 | 1.587 | 88.14272 | 89.27907 | 1.55751 |
| (5 2 3) | 59.34336 | 8.478 | 203.6499 | 206.2754 | 1.55751 |
| (3 6 1) | 59.98778 | 1.118 | 74.72395 | 72.14137 | 1.54229 |
| (6 3 1) | 59.98778 | 1.310 | 80.86849 | 78.07356 | 1.54229 |
| (1 4 4) | 60.28748 | 0.067 | 19.07032 | 14.94373 | 1.53533 |
| (4 1 4) | 60.28748 | 0.645 | 56.47114 | 44.2515 | 1.53533 |
| (0 8 1) | 60.48801 | 0.449 | 47.84614 | 49.88968 | 1.53071 |
| (7 2 0) | 60.56787 | 0.484 | 60.12602 | 58.72625 | 1.52888 |
| (2 7 0) | 60.56787 | 0.357 | 70.16079 | 68.52741 | 1.52888 |
| (1 7 2) | 61.24351 | 6.264 | 180.2055 | 169.7129 | 1.5136 |
| (7 1 2) | 61.24351 | 0.215 | 33.43637 | 31.48952 | 1.5136 |
| (1 6 3) | 61.34229 | 0.042 | 14.64974 | 14.26538 | 1.5114 |
| (6 1 3) | 61.34229 | 0.833 | 65.79574 | 64.06935 | 1.5114 |
| (2 7 1) | 61.97419 | 0.108 | 65.93697 | 36.9678 | 1.49747 |
| (7 2 1) | 61.97419 | 0.823 | 24.07409 | 13.49722 | 1.49747 |
| (0 5 4) | 62.26821 | 0.211 | 34.07138 | 9.498526 | 1.4911 |
| (0 1 5) | 63.03041 | 0.267 | 37.70975 | 25.61168 | 1.47486 |
| (4 5 2) | 63.20666 | 2.029 | 93.63961 | 84.52218 | 1.47117 |
| (5 4 2) | 63.20666 | 1.599 | 105.6244 | 95.34 | 1.47117 |
| (3 3 4) | 63.24499 | 6.405 | 187.7418 | 172.7327 | 1.47037 |
| (1 8 0) | 63.51774 | 1.084 | 109.6136 | 94.89532 | 1.4647 |
| (8 1 0) | 63.51774 | 0.014 | 12.41688 | 10.7496 | 1.4647 |
| (2 4 4) | 63.73022 | 0.330 | 43.13619 | 38.85128 | 1.46032 |
| (4 2 4) | 63.73022 | 0.403 | 47.13927 | 42.45671 | 1.46032 |
| (4 4 3) | 63.78865 | 2.581 | 120.0036 | 106.1001 | 1.45913 |
| (1 1 5) | 64.00064 | 2.363 | 115.2784 | 104.4882 | 1.4548 |
| (3 6 2) | 64.17541 | 1.954 | 104.9416 | 100.9836 | 1.45125 |
| (6 3 2) | 64.17541 | 2.768 | 125.0705 | 120.3533 | 1.45125 |
| (0 7 3) | 64.27161 | 0.954 | 73.49797 | 70.68583 | 1.44931 |
| (3 5 3) | 64.27161 | 0.097 | 23.27054 | 22.38017 | 1.44931 |
| (5 3 3) | 64.27161 | 0.286 | 40.07085 | 38.53768 | 1.44931 |
| (0 2 5) | 64.48273 | 0.296 | 40.83562 | 36.78733 | 1.44507 |
| (5 5 0) | 64.48391 | 0.312 | 59.6689 | 53.63895 | 1.44504 |
| (0 8 2) | 64.6568 | 0.062 | 18.80585 | 12.66479 | 1.44159 |
| (1 8 1) | 64.88732 | 0.067 | 18.16629 | 18.55988 | 1.43702 |
| (8 1 1) | 64.88732 | 0.057 | 19.58757 | 20.01197 | 1.43702 |
| (4 6 0) | 64.96406 | 0.421 | 69.69077 | 62.03955 | 1.4355 |
| (6 4 0) | 64.96406 | 0.367 | 64.97962 | 57.84562 | 1.4355 |
| (1 5 4) | 65.17399 | 0.983 | 75.52607 | 46.40931 | 1.43138 |
| (5 1 4) | 65.17399 | 0.123 | 26.61206 | 16.35261 | 1.43138 |
| (2 6 3) | 65.70895 | 0.657 | 62.18041 | 51.31958 | 1.421 |
| (6 2 3) | 65.70895 | 0.614 | 59.88745 | 49.42713 | 1.421 |
| (5 5 1) | 65.84263 | 0.379 | 46.98808 | 39.70354 | 1.41844 |
| (1 2 5) | 65.91763 | 0.091 | 23.48393 | 19.3472 | 1.417 |
| (2 1 5) | 65.91763 | 0.176 | 32.17561 | 26.50781 | 1.417 |
| (2 7 2) | 66.08969 | 0.161 | 30.84508 | 26.79028 | 1.41372 |
| (7 2 2) | 66.08969 | 1.492 | 94.06566 | 81.7001 | 1.41372 |
| (4 6 1) | 66.31759 | 0.251 | 38.60793 | 35.35114 | 1.40941 |
| (6 4 1) | 66.31759 | 0.519 | 55.70946 | 51.01008 | 1.40941 |
| (3 7 0) | 66.39347 | 0.043 | 22.77378 | 17.81859 | 1.40798 |
| (7 3 0) | 66.39347 | 0.162 | 44.28855 | 34.65207 | 1.40798 |
| (0 3 5) | 66.86528 | 4.480 | 164.865 | 153.2898 | 1.39918 |
| (0 9 0) | 67.33772 | 3.069 | 194.1243 | 182.1174 | 1.39049 |
| (0 6 4) | 67.54384 | 0.071 | 20.96044 | 16.1921 | 1.38674 |
| (3 7 1) | 67.73231 | 0.244 | 38.87627 | 25.07353 | 1.38334 |
| (7 3 1) | 67.73231 | 0.738 | 67.61211 | 43.60693 | 1.38334 |
| (3 4 4) | 68.01282 | 0.034 | 14.52808 | 11.95713 | 1.37831 |
| (4 3 4) | 68.01282 | 0.762 | 69.0727 | 56.84934 | 1.37831 |
| (7 1 3) | 68.06931 | 0.654 | 63.94663 | 57.5173 | 1.3773 |
| (1 7 3) | 68.06931 | 0.436 | 52.19583 | 46.94795 | 1.3773 |
| (2 2 5) | 68.27436 | 4.113 | 160.775 | 154.2006 | 1.37366 |
| (0 9 1) | 68.66748 | 1.543 | 99.07156 | 70.23567 | 1.36674 |
| (1 3 5) | 68.74093 | 0.110 | 25.74853 | 20.45654 | 1.36546 |
| (3 1 5) | 68.74093 | 0.499 | 56.29506 | 44.72494 | 1.36546 |
| (2 8 0) | 68.74207 | 0.093 | 31.93262 | 25.405 | 1.36544 |
| (8 2 0) | 68.74207 | 0.080 | 34.69643 | 27.60382 | 1.36544 |
| (8 1 2) | 68.90949 | 0.048 | 17.4745 | 16.51418 | 1.36252 |
| (1 8 2) | 68.90949 | 0.114 | 26.94021 | 25.45967 | 1.36252 |
| (2 5 4) | 68.94617 | 0.421 | 52.06853 | 48.78513 | 1.36189 |
| (5 2 4) | 68.94617 | 0.594 | 61.59071 | 57.70685 | 1.36189 |
| (5 5 2) | 69.83722 | 1.417 | 96.10966 | 78.47927 | 1.34665 |
| (4 5 3) | 69.92946 | 0.252 | 17.81828 | 13.23261 | 1.3451 |
| (5 4 3) | 69.92946 | 0.047 | 40.57404 | 30.13201 | 1.3451 |
| (2 8 1) | 70.05918 | 0.430 | 38.76109 | 29.8327 | 1.34292 |
| (8 2 1) | 70.05918 | 0.229 | 53.05389 | 40.83323 | 1.34292 |
| (0 4 5) | 70.13197 | 0.382 | 50.07542 | 40.41873 | 1.34171 |
| (4 6 2) | 70.299 | 1.259 | 91.08841 | 77.58785 | 1.33892 |
| (6 4 2) | 70.299 | 0.165 | 32.90954 | 28.03189 | 1.33892 |
| (1 6 4) | 70.79568 | 0.238 | 39.87682 | 38.57322 | 1.33073 |
| (6 1 4) | 70.79568 | 0.515 | 58.67595 | 56.75779 | 1.33073 |
| (6 3 3) | 70.85115 | 0.677 | 67.17464 | 64.44185 | 1.32982 |
| (3 6 3) | 70.85115 | 1.218 | 90.18444 | 86.51557 | 1.32982 |
| (0 8 3) | 71.31005 | 0.468 | 56.16187 | 30.99532 | 1.32238 |
| (2 3 5) | 71.51085 | 0.465 | 56.22134 | 40.91149 | 1.31915 |
| (3 2 5) | 71.51085 | 0.047 | 17.25923 | 12.5593 | 1.31915 |
| (3 7 2) | 71.67649 | 0.632 | 65.56085 | 59.17021 | 1.31651 |
| (7 3 2) | 71.67649 | 0.009 | 7.815689 | 7.053864 | 1.31651 |
| (1 9 0) | 71.96908 | 1.313 | 134.0969 | 119.3491 | 1.31187 |
| (5 6 0) | 71.96908 | 0.077 | 36.17405 | 32.19567 | 1.31187 |
| (6 5 0) | 71.96908 | 0.096 | 59.27118 | 52.75261 | 1.31187 |
| (9 1 0) | 71.96908 | 0.257 | 32.63486 | 29.04572 | 1.31187 |
| (1 4 5) | 72.42389 | 4.956 | 185.1822 | 166.26 | 1.30474 |
| (4 1 5) | 72.42389 | 1.016 | 83.89331 | 75.32099 | 1.30474 |
| (0 9 2) | 72.58868 | 0.195 | 36.72072 | 34.11105 | 1.30218 |
| (2 7 3) | 72.67942 | 0.123 | 29.44926 | 27.34008 | 1.30077 |
| (7 2 3) | 72.67942 | 0.085 | 24.0161 | 22.29605 | 1.30077 |
| (7 4 0) | 72.87978 | 0.488 | 82.49109 | 91.91777 | 1.29769 |
| (4 7 0) | 72.87978 | 0.290 | 63.67786 | 70.95465 | 1.29769 |
| (4 4 4) | 73.07882 | 0.332 | 48.34834 | 44.82792 | 1.29464 |
| (9 1 1) | 73.26088 | 0.030 | 14.45967 | 12.59956 | 1.29187 |
| (1 9 1) | 73.26088 | 0.728 | 71.64962 | 62.4326 | 1.29187 |
| (5 6 1) | 73.26088 | 0.020 | 11.90878 | 10.37685 | 1.29187 |
| (6 5 1) | 73.26088 | 0.130 | 30.17177 | 26.29047 | 1.29187 |
| (0 7 4) | 73.532 | 0.241 | 41.30013 | 32.22791 | 1.28776 |
| (3 5 4) | 73.532 | 0.153 | 32.7585 | 25.56259 | 1.28776 |
| (5 3 4) | 73.532 | 0.743 | 72.55218 | 56.61495 | 1.28776 |
| (8 2 2) | 73.94851 | 2.721 | 139.4189 | 125.5712 | 1.28153 |
| (2 8 2) | 73.94851 | 0.054 | 19.69043 | 17.73471 | 1.28153 |
| (4 7 1) | 74.16533 | 1.851 | 115.1584 | 112.759 | 1.27831 |
| (7 4 1) | 74.16533 | 0.182 | 36.15198 | 35.39874 | 1.27831 |
| (0 5 5) | 74.23645 | 0.260 | 43.31243 | 36.27538 | 1.27726 |
| (3 8 0) | 74.68808 | 0.095 | 37.02634 | 13.10626 | 1.27065 |
| (8 3 0) | 74.68808 | 0.187 | 52.38914 | 18.54422 | 1.27065 |
| (2 6 4) | 74.8853 | 0.184 | 36.34503 | 13.64386 | 1.26779 |
| (6 2 4) | 74.8853 | 0.133 | 31.78614 | 11.93248 | 1.26779 |
| (3 3 5) | 75.13654 | 0.918 | 81.93789 | 38.82981 | 1.26416 |
| (1 8 3) | 75.38859 | 0.011 | 47.75575 | 32.38822 | 1.26056 |
| (8 1 3) | 75.38859 | 0.312 | 9.207225 | 6.244357 | 1.26056 |
| (2 4 5) | 75.58516 | 0.053 | 19.98327 | 8.327725 | 1.25776 |
| (4 2 5) | 75.58516 | 0.160 | 33.72192 | 14.05308 | 1.25776 |
| (3 8 1) | 75.96235 | 1.035 | 87.85787 | 43.23375 | 1.25245 |
| (8 3 1) | 75.96235 | 0.050 | 19.39283 | 9.542956 | 1.25245 |
| (0 10 0) | 76.03397 | 0.001 | 3.082531 | 1.792205 | 1.25144 |
| (5 5 3) | 76.28391 | 0.538 | 63.3549 | 38.00116 | 1.24796 |
| (4 6 3) | 76.73027 | 0.291 | 46.91383 | 23.65652 | 1.2418 |
| (6 4 3) | 76.73027 | 0.316 | 48.7246 | 24.56964 | 1.2418 |
| (1 5 5) | 76.92571 | 0.233 | 41.56352 | 35.11688 | 1.23913 |
| (5 1 5) | 76.92571 | 0.073 | 23.32854 | 19.7102 | 1.23913 |
| (0 0 6) | 77.03215 | 2.291 | 301.2056 | 0.126491 | 1.23768 |
| (1 9 2) | 77.08699 | 0.065 | 22.25163 | 20.75114 | 1.23693 |
| (5 6 2) | 77.08699 | 0.920 | 38.24547 | 35.6665 | 1.23693 |
| (6 5 2) | 77.08699 | 0.005 | 83.54403 | 77.91046 | 1.23693 |
| (9 1 2) | 77.08699 | 0.192 | 5.978545 | 5.575392 | 1.23693 |
| (1 7 4) | 77.12209 | 0.218 | 99.00975 | 91.27071 | 1.23646 |
| (7 1 4) | 77.12209 | 1.293 | 40.93271 | 37.73323 | 1.23646 |
| (0 10 1) | 77.30077 | 0.031 | 15.48231 | 12.00862 | 1.23404 |
| (2 9 0) | 77.37199 | 0.509 | 88.32805 | 61.56868 | 1.23308 |
| (9 2 0) | 77.37199 | 0.124 | 43.64431 | 30.42208 | 1.23308 |
| (0 1 6) | 77.47715 | 0.059 | 20.73036 | 11.96382 | 1.23167 |
| (4 7 2) | 77.97603 | 4.083 | 32.30941 | 30.28846 | 1.22502 |
| (7 4 2) | 77.97603 | 0.135 | 177.4698 | 166.3691 | 1.22502 |
| (3 7 3) | 78.06455 | 0.022 | 12.90512 | 11.93482 | 1.22386 |
| (7 3 3) | 78.06455 | 0.057 | 21.2826 | 19.6824 | 1.22386 |
| (1 1 6) | 78.36487 | 0.429 | 57.73999 | 40.81087 | 1.21991 |
| (9 2 1) | 78.63214 | 0.312 | 49.35638 | 45.64602 | 1.21643 |
| (2 9 1) | 78.63214 | 0.393 | 55.35163 | 51.19058 | 1.21643 |
| (0 2 6) | 78.80765 | 0.200 | 39.32429 | 32.69766 | 1.21416 |
| (4 5 4) | 78.89699 | 0.131 | 31.65222 | 24.51916 | 1.213 |
| (5 4 4) | 78.89699 | 0.464 | 60.59259 | 46.93759 | 1.213 |
| (0 9 3) | 78.95036 | 0.043 | 18.20091 | 14.07476 | 1.21232 |
| (0 6 5) | 79.14418 | 0.022 | 14.21914 | 8.955669 | 1.20983 |
| (3 4 5) | 79.58578 | 0.065 | 22.78359 | 19.82456 | 1.20421 |
| (4 3 5) | 79.58578 | 0.014 | 10.84518 | 9.436684 | 1.20421 |
| (6 6 0) | 79.58686 | 3.101 | 221.5294 | 193.0179 | 1.2042 |
| (3 8 2) | 79.7455 | 0.260 | 45.53987 | 42.80895 | 1.2022 |
| (8 3 2) | 79.7455 | 1.113 | 93.99846 | 88.36158 | 1.2022 |
| (3 6 4) | 79.78027 | 0.246 | 44.24112 | 42.18322 | 1.20176 |
| (6 3 4) | 79.78027 | 0.176 | 37.58158 | 35.83345 | 1.20176 |
| (5 7 0) | 80.02782 | 0.524 | 91.59565 | 87.62762 | 1.19866 |
| (7 5 0) | 80.02782 | 1.101 | 132.5744 | 126.8311 | 1.19866 |
| (1 2 6) | 80.13202 | 0.219 | 41.27224 | 39.67032 | 1.19737 |
| (2 1 6) | 80.13202 | 0.018 | 12.39105 | 11.91008 | 1.19737 |
| (0 8 4) | 80.22097 | 0.084 | 25.61853 | 25.35928 | 1.19626 |
| (8 2 3) | 80.27411 | 0.205 | 32.03974 | 31.76983 | 1.1956 |
| (2 8 3) | 80.27411 | 0.127 | 40.62998 | 40.2877 | 1.1956 |
| (2 5 5) | 80.46712 | 0.432 | 58.97066 | 55.85449 | 1.19322 |
| (5 2 5) | 80.46712 | 0.705 | 75.18724 | 71.21415 | 1.19322 |
| (6 6 1) | 80.83761 | 0.067 | 23.23091 | 13.58415 | 1.18867 |
| (1 10 0) | 80.90799 | 0.059 | 30.80901 | 19.17045 | 1.18782 |
| (10 1 0) | 80.90799 | 0.318 | 71.45348 | 44.46083 | 1.18782 |
| (0 3 6) | 81.01191 | 0.529 | 66.16581 | 48.30195 | 1.18655 |
| (0 10 2) | 81.06601 | 0.449 | 60.04473 | 46.96981 | 1.1859 |
| (5 7 1) | 81.27695 | 0.382 | 55.72154 | 42.46576 | 1.18335 |
| (7 5 1) | 81.27695 | 0.032 | 16.05114 | 12.2327 | 1.18335 |
| (4 8 0) | 81.34724 | 1.917 | 176.5804 | 127.3249 | 1.1825 |
| (8 4 0) | 81.34724 | 0.207 | 58.17395 | 41.94684 | 1.1825 |
| (2 7 4) | 81.53966 | 0.022 | 13.83445 | 7.18568 | 1.18019 |
| (7 2 4) | 81.53966 | 0.213 | 41.70861 | 21.66368 | 1.18019 |
| (1 10 1) | 82.15409 | 0.361 | 54.44472 | 40.4024 | 1.17291 |
| (10 1 1) | 82.15409 | 0.183 | 38.84609 | 28.82697 | 1.17291 |
| (1 6 5) | 82.22315 | 0.109 | 30.31336 | 23.68749 | 1.1721 |
| (6 1 5) | 82.22315 | 0.396 | 57.06372 | 44.59076 | 1.1721 |
| (2 2 6) | 82.32779 | 0.102 | 28.97737 | 21.63218 | 1.17087 |
| (2 9 2) | 82.3817 | 0.047 | 19.69188 | 14.57042 | 1.17024 |
| (9 2 2) | 82.3817 | 0.638 | 72.6575 | 53.76071 | 1.17024 |
| (4 8 1) | 82.59194 | 0.300 | 29.70253 | 19.89832 | 1.16779 |
| (8 4 1) | 82.59194 | 0.107 | 50.02048 | 33.50973 | 1.16779 |
| (1 3 6) | 82.76547 | 0.436 | 60.09714 | 33.99228 | 1.16578 |
| (3 1 6) | 82.76547 | 0.403 | 57.80095 | 32.69352 | 1.16578 |
| (9 1 3) | 83.34371 | 0.697 | 76.44996 | 63.17422 | 1.15915 |
| (1 9 3) | 83.34371 | 0.724 | 63.90843 | 52.81057 | 1.15915 |
| (5 6 3) | 83.34371 | 0.488 | 12.63329 | 10.43949 | 1.15915 |
| (6 5 3) | 83.34371 | 0.019 | 78.01953 | 64.47123 | 1.15915 |
| (3 9 0) | 83.5363 | 0.964 | 127.2236 | 83.23297 | 1.15696 |
| (9 3 0) | 83.5363 | 0.109 | 42.92411 | 28.08206 | 1.15696 |
| (0 4 6) | 84.07604 | 0.219 | 43.36195 | 29.43299 | 1.15089 |
| (1 8 4) | 84.16415 | 0.004 | 73.91809 | 62.07442 | 1.14991 |
| (8 1 4) | 84.16415 | 0.645 | 5.638617 | 4.735187 | 1.14991 |
| (4 7 3) | 84.21679 | 1.371 | 107.8106 | 97.4636 | 1.14933 |
| (7 4 3) | 84.21679 | 0.509 | 65.8276 | 59.50986 | 1.14933 |
| (4 4 5) | 84.408 | 0.200 | 40.44857 | 33.98997 | 1.14721 |
| (6 6 2) | 84.56584 | 0.031 | 16.2681 | 13.19644 | 1.14547 |
| (3 9 1) | 84.77518 | 0.006 | 6.845509 | 6.032081 | 1.14317 |
| (9 3 1) | 84.77518 | 0.684 | 76.42717 | 67.3454 | 1.14317 |
| (0 7 5) | 84.84387 | 0.292 | 49.89448 | 41.47083 | 1.14242 |
| (3 5 5) | 84.84387 | 0.336 | 53.61272 | 44.56133 | 1.14242 |
| (5 3 5) | 84.84387 | 0.125 | 32.63058 | 27.12158 | 1.14242 |
| (5 7 2) | 85.00161 | 0.479 | 11.32241 | 8.03567 | 1.1407 |
| (7 5 2) | 85.00161 | 0.015 | 64.19048 | 45.55685 | 1.1407 |
| (5 5 4) | 85.03595 | 0.110 | 30.75391 | 20.86849 | 1.14032 |
| (0 11 0) | 85.28051 | 0.608 | 102.3897 | 59.74228 | 1.13768 |
| (2 3 6) | 85.38348 | 0.233 | 44.59171 | 23.67615 | 1.13657 |
| (3 2 6) | 85.38348 | 0.172 | 38.12444 | 20.24233 | 1.13657 |
| (4 6 4) | 85.47139 | 0.031 | 16.45351 | 7.979348 | 1.13562 |
| (6 4 4) | 85.47139 | 0.030 | 16.29521 | 7.902595 | 1.13562 |
| (1 10 2) | 85.87228 | 0.086 | 27.11902 | 19.45531 | 1.13134 |
| (10 1 2) | 85.87228 | 0.546 | 68.80295 | 49.35956 | 1.13134 |
| (3 8 3) | 85.95908 | 0.035 | 17.48414 | 14.34008 | 1.13042 |
| (8 3 3) | 85.95908 | 1.215 | 102.6784 | 84.21443 | 1.13042 |
| (2 6 5) | 86.14979 | 0.073 | 25.33478 | 21.90123 | 1.1284 |
| (6 2 5) | 86.14979 | 0.146 | 35.78754 | 30.93734 | 1.1284 |
| (1 4 6) | 86.2537 | 0.155 | 36.95973 | 33.91519 | 1.1273 |
| (4 1 6) | 86.2537 | 1.493 | 113.7846 | 104.4116 | 1.1273 |
| (4 8 2) | 86.30724 | 0.174 | 42.98189 | 40.24885 | 1.12674 |
| (8 4 2) | 86.30724 | 0.212 | 38.82481 | 36.3561 | 1.12674 |
| (0 11 1) | 86.51607 | 0.011 | 9.54217 | 8.63684 | 1.12455 |
| (10 2 0) | 86.58567 | 0.070 | 35.01057 | 29.85245 | 1.12383 |
| (2 10 0) | 86.58567 | 0.356 | 78.88555 | 67.26336 | 1.12383 |
| (3 7 4) | 86.77625 | 0.271 | 48.64306 | 38.36764 | 1.12185 |
| (7 3 4) | 86.77625 | 0.217 | 43.51432 | 34.32231 | 1.12185 |
| (0 10 3) | 87.26321 | 1.613 | 119.0048 | 96.44971 | 1.11683 |
| (0 9 4) | 87.64516 | 2.435 | 146.6268 | 117.6229 | 1.11294 |
| (2 10 1) | 87.8195 | 0.150 | 36.52746 | 32.65645 | 1.11118 |
| (10 2 1) | 87.8195 | 0.341 | 54.77341 | 48.96881 | 1.11118 |
| (6 7 0) | 87.88902 | 0.107 | 43.39175 | 39.00992 | 1.11048 |
| (7 6 0) | 87.88902 | 0.584 | 101.5607 | 91.3048 | 1.11048 |
| (0 5 6) | 87.99171 | 0.058 | 23.05528 | 19.61905 | 1.10944 |
| (1 7 5) | 88.32213 | 0.903 | 89.48563 | 81.91504 | 1.10614 |
| (7 1 5) | 88.32213 | 2.454 | 147.8136 | 135.3084 | 1.10614 |
| (3 9 2) | 88.47929 | 0.421 | 61.19032 | 58.65345 | 1.10458 |
| (9 3 2) | 88.47929 | 0.946 | 91.7978 | 87.992 | 1.10458 |
| (2 9 3) | 88.56589 | 0.012 | 10.19833 | 9.134878 | 1.10372 |
| (9 2 3) | 88.56589 | 0.036 | 17.95876 | 16.08608 | 1.10372 |
| (5 8 0) | 88.75726 | 0.078 | 15.7489 | 12.48859 | 1.10183 |
| (8 5 0) | 88.75726 | 0.013 | 37.48124 | 29.72193 | 1.10183 |
| (3 3 6) | 88.85989 | 1.367 | 110.2985 | 91.24542 | 1.10083 |
| (8 2 4) | 88.94754 | 0.780 | 83.62323 | 66.42694 | 1.09997 |
| (2 8 4) | 88.94754 | 0.536 | 69.45535 | 55.17254 | 1.09997 |
| (6 7 1) | 89.12177 | 0.272 | 49.48936 | 30.46944 | 1.09826 |
| (7 6 1) | 89.12177 | 0.161 | 38.16235 | 23.49568 | 1.09826 |
| (2 4 6) | 89.29387 | 0.107 | 31.11109 | 17.80514 | 1.09659 |
| (4 2 6) | 89.29387 | 0.174 | 38.71851 | 22.15893 | 1.09659 |
| (5 8 1) | 89.98964 | 0.220 | 44.49147 | 31.31126 | 1.0899 |
| (8 5 1) | 89.98964 | 0.329 | 54.7514 | 38.53177 | 1.0899 |

Table S6. Calculated atoms’ magnetic moments of Fe_7_P_11_O_38_ phase.

| Atom | Magnetic moment (µ_B_) |
| --- | --- |
| Interstitial magnetic moment | -0.03513 |
| Fe1 | 4.10431 |
| Fe2 | -4.14410 |
| Fe3 | -0.85682 |
| P1 | -0.00497 |
| P2 | -0.00737 |
| P3 | -0.00189 |
| P4 | -0.00039 |
| P5 | -0.00322 |
| O1 | -0.00338 |
| O2 | 0.00580 |
| O3 | -0.00670 |
| O4 | -0.07120 |
| O5 | 0.07963 |
| O6 | -0.08223 |
| O7 | 0.04972 |
| O8 | -0.07969 |
| O9 | 0.10016 |
| O10 | -0.00136 |
| O12 | 0.02573 |
| O13 | 0.00120 |
| O14 | -0.01222 |
| Total spin magnetic moment in cell | -2.00049 |

Table S7. Selected critic points for new Fe_7_P_11_O_38_ phase (electron density $\rho$, Laplacian $\nabla^{2}\rho$, electrostatic potential $V,$ and kinetic $G$ energies).

| fragment of structure | i | j | $\rho(e/Å^{3} )$ | $\nabla^{2}\rho(e/Å^{5} )$ | $G (au)$ | $V (au)$ | $\left\vert V \right\vert/G$ |
| --- | --- | --- | --- | --- | --- | --- | --- |
| [Fe1O_6_]-[Fe2O_6_] | Fe1 | O2 | 0.059 | 0.226 | 0.067 | -0.074 | 1.110 |
|  | Fe2 | O2 | 0.050 | 0.175 | 0.049 | -0.054 | 1.103 |
|  | Fe1 | O1 | 0.061 | 0.238 | 0.067 | -0.074 | 1.110 |
|  | Fe2 | O2 | 0.061 | 0.240 | 0.067 | -0.075 | 1.110 |
|  | Fe1 | O3 | 0.053 | 0.193 | 0.054 | -0.059 | 1.105 |
|  | Fe2 | O3 | 0.069 | 0.288 | 0.081 | -0.091 | 1.114 |
|  | Fe1 | O9 | 0.108 | 0.550 | 0.162 | -0.186 | 1.150 |
|  | Fe1 | O7 | 0.085 | 0.393 | 0.112 | -0.126 | 1.124 |
|  | Fe1 | O5 | 0.009 | 0.422 | 0.121 | -0.137 | 1.128 |
|  | Fe2 | O6 | 0.107 | 0.549 | 0.161 | -0.185 | 1.149 |
|  | Fe2 | O8 | 0.099 | 0.488 | 0.142 | -0.161 | 1.140 |
|  | Fe2 | O4 | 0.097 | 0.479 | 0.139 | -0.158 | 1.138 |
|  | cave critic point on  Fe1-Fe2 connection | | 0.027 | 0.054 | 0.016 | -0.018 | 1.156 |
|  | ring critic points | | 0.028 | 0.075 | 0.020 | -0.021 | 1.067 |
|  |  |  | 0.028 | 0.063 | 0.018 | -0.020 | 1.118 |
|  |  |  | 0.027 | 0.058 | 0.017 | -0.019 | 1.127 |
| [Fe3O_4_]-[PO_4_] | Fe3 | O11 | 0.153 | 0.866 | 0.271 | -0.325 | 1.200 |
|  | Fe3 | O14 | 0.142 | 0.243 | 0.243 | -0.289 | 1.188 |
|  | P6 | O14 | 0.179 | 0.651 | 0.271 | -0.380 | 1.401 |
|  | P6 | O10 | 0.186 | 0.786 | 0.306 | -0.415 | 1.357 |
|  | P6 | Fe3 | 0.005 | 0.010 | 0.002 | -0.002 | 0.810 |
